# Global, regional, and national impact of epidemic disasters on health workforce equality between 1990 and 2019: An ecological and modeling study

**DOI:** 10.1371/journal.pmed.1005134

**Published:** 2026-09-22

**Authors:** Manman Chen, Wanzhou Wang, Feng Sha, Zichen Ye, Yuankai Zhao, Chao Yang, Ze Liang, Yu Jiang, Xijie Wang, Jinling Tang

**Affiliations:** 1 School of Population Medicine and Public Health, Chinese Academy of Medical Sciences & Peking Union Medical College, Beijing, China; 2 National Institute of Health Data Science, Peking University, Beijing, China; 3 Center for Digital Health and Artificial Intelligence, Peking University First Hospital, Beijing, China; 4 Department of Computational Biology and Medical Big Data, Faculty of Computer Science and Artificial Intelligence, Shenzhen University of Advanced Technology, Shenzhen, Guangdong, China; 5 Shenzhen Institute of Advanced Technology, Chinese Academy of Sciences, Shenzhen, Guangdong, China; 6 School of Health Policy and Management, Chinese Academy of Medical Sciences & Peking Union Medical College, Beijing, China; 7 Renal Division, Department of Medicine, Peking University First Hospital, Peking University Institute of Nephrology, Beijing, China; 8 School of Economics and Management, Harbin Institute of Technology, Harbin, China; 9 School of Disaster and Emergency Medicine, Tianjin University, Tianjin, China; Washington University In St Louis: Washington University in St Louis, UNITED STATES OF AMERICA

## Abstract

**Background:**

Global distribution of human resources for health (HRH) is highly unequal and less resourced regions may particularly be vulnerable to disastrous emergencies. We examined HRH losses attributable to epidemics of infectious diseases, both globally and geographically.

**Methods and findings:**

First, using 30-year ecological data from the 1990 to 2019 records at international Emergency Events Database and HRH density statistics from the Global Burden of Disease study, we extracted estimates of annual country-specific epidemic events and cadre-specific HRH density data across 194 countries and territories. The association between the number of epidemics and HRH density (number of HRH per 10,000 population) was established using the Generalized Estimating Equation model, adjusted for geographic and socioeconomic status. Second, based on the country- and year-specific number of epidemics, HRH density, and epidemic-HRH density associations, we further estimated the annual average absolute number of HRH losses and the average relative number of HRH losses per 10,000 HRHs that can be attributed to epidemic events, globally, by region, and by country.

From 1990 to 2019, 1,185 epidemic events were recorded in 194 countries and regions. Globally, an increase in 1 epidemic event in the preceding year was associated with an average decrease of 57.5 × 10^−3^ (95% confidence interval (CI) [18.5, 96.4]; *p* = 0.004) in HRH density in the following year. Assuming the observed epidemic-HRH associations were causal, we estimated that globally an average loss of 17,549 (95% CI [5,661, 29,437]) HRHs, or 2.57 (95% CI [0.83, 4.31]) per 10,000 HRHs were attributable to epidemics each year. During the entire observation period, South Asia and sub-Saharan Africa had the highest average attributable fractions, at 20.52 and 18.03 per 10,000, respectively. Low- (15.89 per 10,000) and lower-middle-income (12.76 per 10,000) countries showed higher average attributable fractions than upper-middle- (0.96 per 10,000) and high-income countries (0.12 per 10,000). The top 10 countries with the highest average attributable fractions were observed mostly in sub-Saharan Africa, such as Niger (57.06 per 10,000), Somalia (44.19 per 10,000), and Ethiopia (37.12 per 10,000). The results may be biased due to uncontrolled confounders and reverse causation, and potential underestimation in countries with weaker disaster-surveillance systems.

**Conclusions:**

Epidemics of infectious diseases are associated with HRH losses globally, particularly in low- and middle-income countries, further aggravating the global inequity in the health workforce.

## Author summary

**Why was this study done?:**

- Health workers are unevenly distributed around the world, and countries with the fewest health workers may be least able to absorb the shock of an infectious disease outbreak.
- Short-term links between epidemics and health workforce strain have been reported before, but no study had quantified how many health workers are lost to epidemics at the global, regional, and national levels, or whether these losses are unequal among countries.
- Understanding this burden could help direct support toward the health workforce protection and epidemic preparedness, particularly in countries that can least afford further losses.

**What did the researchers do and find?:**

- The researchers combined 30 years (1990–2019) of data on epidemic disasters from the emergency event records with health worker density data for 16 types of health worker across 194 countries and territories.
- Using statistical models that adjusted for a country’s income level, socio-demographic development, urbanization, and population density, this study found that an outbreak of epidemics in a country was followed by a fall in health worker density in the subsequent year.
- Assuming these associations were causal, this study estimated that epidemics were associated with an average loss of around 17,500 health workers globally each year, and low- and lower-middle-income countries experienced disproportionately greater losses.

**What do these findings mean?:**

- Epidemics may be an underappreciated and unequally distributed cause of health workforce losses, compounding pre-existing shortages in countries that already have too few health workers.
- Plans to prepare for future epidemics could usefully include specific measures to protect and retain health workers, especially nurses, midwives, and physicians, especially in lower-resource settings.
- Relying on country-level rather than individual-level of data, the study cannot fully rule out other explanations, such as reverse causation, migration, or endemic diseases, and may underestimate losses in countries with weak disaster-reporting systems.

## Abstract 3

Manman Chen, Wanzhou Wang and colleagues examined how infectious disease epidemics were associated with changes in the health workforce across 194 countries and territories from 1990 to 2019. Their findings suggest that epidemic events were linked to health workforce losses worldwide, with the largest relative impacts observed in low- and middle-income countries.

## Introduction

Human resources for health (HRH), namely all people engaged in work that aims chiefly to improve health, is a fundamental pillar of health systems and essential for achieving universal health coverage [1,2]. Optimizing the management of the HRH is necessary for the progressive realization of universal health coverage [3,4]. One of the specific targets of the Sustainable Development Goals 3 (SDG target 3.C) is to substantially increase health financing and the recruitment, development, training, and retention of the health workforce in developing countries, especially in less developed countries and small island developing states [5]. However, there are still profound inequalities in the global distribution of the health workforce [6,7]. United Nations’ statistics from 2014 to 2020 reveal that, despite a steady global increase in medical doctor density, the disparity among regions ranges from an estimated 40 physicians per 10,000 people in Europe to only 2 in sub-Saharan Africa [8], and the disparities between high-income and low-income countries also reached 6.5 fold [9]. Projections indicate that by 2030, the shortage of the global HRH will decrease by 33%, while that in African and eastern Mediterranean regions will decline by only 7% and 15% [9].

The stability and capacity of HRH fundamentally determine epidemic preparedness and health system resilience [10,11]. As the frontline defense against global health threats, such as Zika viruses, Middle East Respiratory Syndrome (MERS), and pandemic influenza, healthcare workers face direct mortality risks during epidemic outbreaks [12]. For instance, during the first year of the coronavirus disease 2019 (COVID-19) pandemic (January 2020 and May 2021), an estimated 115,500 healthcare workers died globally [8]. Beyond immediate fatalities, epidemics may trigger systemic attrition through economic contraction, reducing healthcare demand and institutional capacity to retain pre-pandemic staffing levels, or occupational disillusionment diminishing clinicians’ willingness to remain in the workforce [13]. Studies have also reported stable aggregate retention during COVID-19’s initial phase [9]. Notably, while the associations between short-term workforce losses and epidemics are documented, existing evidence fails to establish the dose–response associations and quantify the HRH losses attributable to epidemics, especially their spatiotemporal characteristics across geographic and socioeconomic contexts. The extent to which epidemics are associated to HRH losses at the global, regional, and national levels remains unknown. Further real-world empirical evidence is imperative to inform targeted recommendations for building resilient HRH systems capable of enhancing epidemic preparedness, particularly in vulnerable regions with constrained healthcare workforces.

This study aims to (1) investigate the exposure-response associations between epidemics and country-level HRHs, (2) estimate the HRH losses attributable to epidemics at the global, regional, and national levels during the past three decades, and (3) comprehensively investigate the global patterns, trends, and inequalities in HRH losses across different geographic and socioeconomic contexts. Based on the evidence above, we hypothesized that epidemics were negatively associated with HRH density, with disproportionately larger losses concentrated in lower Socio-demographic Index (SDI) and lower-income settings already facing the greatest HRH shortages. To address these critical knowledge gaps and test these hypotheses, this study performed a systematic analysis based on the Emergency Events Database (EM-DAT) and the most up-to-date available statistics on the global HRH.

## Methods

### Study overview

This study is reported as per the Reporting of Studies Conducted using Observational Routinely-Collected Data (RECORD) guideline (S1 Checklist) and the Guidelines for Accurate and Transparent Health Estimates Reporting (GATHER) statement (S2 Checklist). Our study did not have a formal prospective analysis plan, but all analyses were planned prior to analyzing the data, as shown in Texts A–C in S1 Appendix. Nevertheless, in response to the reviewer comments during the peer review process, we conducted additional sensitivity analyses to evaluate potential reverse causation, account for epidemic intensity, and control for potential confounding from baseline disease burden, socioeconomic status, and long-term temporal trends. This study followed a two-stage approach. In the first stage, we established exposure-response associations between epidemic disasters and HRH using a longitudinal ecological design by generating a country-year panel dataset covering 194 countries and territories from 1990 to 2019. In the second stage, we quantified epidemic-attributable HRH losses. The analyses further evaluated inequality in epidemic-attributable HRH losses across countries with different income levels, sociodemographic contexts, and geographic locations.

### Data sources and processing

Annual counts of epidemic events were obtained from the EM-DAT, maintained by the Centre for Research on the Epidemiology of Disasters [14], The EM-DAT includes only major disaster events meeting at least one of the following country-level criteria: (1) 10 or more reported deaths; (2) at least 100 individuals affected; (3) a declared state of emergency; and/or (4) a formal international assistance request. EM-DAT data are compiled from multiple sources, including United Nations agencies, non-governmental organizations, reinsurance companies, research institutes, and media reports [14]. For each country-year between 1990 and 2019, we calculated the total number of epidemic events. The final dataset included 1,185 epidemic events in 132 out of 194 countries and territories (62 had no records) from 1990 to 2019 [15].

HRH density data were derived from a published dataset on country-level cadre-specific HRH from 1990 to 2019, which compiled national HRH estimates using labor-force surveys, census data, and World Health Organization (WHO)’s National Health Workforce Accounts (NHWA) [16]. Occupational responses were harmonized using the International Standard Classification of Occupations (ISCO‑88) at the 3- or 4-digit level [16,17]. Sixteen health worker cadres were identified and harmonized across data sources, with adjustments made using lasso-regression-derived crosswalks to account for reporting inconsistencies. The cadre-specific data were then internally rescaled so that the sum of all cadres equaled the total HRH estimate for each country and year. The included HRH cadres were: All health workers (total HRH), Aides & Emergency Medical Workers, Audiologists and Counsellors, Dentistry personnel, Dieticians & Nutritionists, Environmental Health Officers, Medical Assistants & Community Health Workers (CHWs), Medical Lab Technicians, Nursing and midwifery personnel, Optometrists, Personal Care Workers, Pharmaceutical personnel, Physicians (Doctors), Psychologists, Radiographers, and Traditional & Complementary Practitioners. All HRH densities were expressed as the number of cadre-specific health workers per 10,000 population [2].

Several covariates were included to adjust for potential confounding factors. The urban population proportion and population density were obtained from the World Bank (https://www.worldbank.org/). Countries were categorized by income group (low, lower-middle, upper-middle, and high income) according to the World Bank’s annual classifications. The SDI, provided by the Global Burden of Disease (GBD) study, is a composite index scaled from 0 to 1, combining income per capita, average educational attainment, and total fertility rate among women under 25. Countries were categorized into five SDI subgroups (low, low-middle, middle, high-middle, and high) [2]. Both the World Bank income classification and the SDI subgroup were collected on a country-year basis. Countries were further classified into seven GBD super-regions and 21 GBD regions based on geographical proximity and epidemiological profiles. We also included the background communicable disability-adjusted life-years (DALYs) burden gross domestic product (GDP) per capita, derived from the GBD study and World Bank, respectively [18]. Detailed information on the data sources and variables is provided in Table A in S1 Appendix.

### Statistical analysis

We summarized the epidemic events and HRH densities by country and year. Temporal trends were analyzed using log-linear regression models to estimate the annual percentage changes. The annual percent change was calculated using the estimated slope.

A panel dataset was constructed using annual observations for each country from 1990 to 2019. Missing values in the time-varying covariates were addressed using linear interpolation. To estimate the association between epidemic events and HRH density while accounting for intra-country correlation over time, we employed generalized estimating equation (GEE) models with an autoregressive (AR1) correlation structure [19]. In country-year panel data, annual observations within the same country are serially correlated, with values in adjacent years more similar than values separated by longer intervals. The AR1 structure can accommodate this by allowing within-country correlation, providing a more appropriate specification for longitudinal panel data [19,20]. The quasi-likelihood information criterion (QIC) further indicated a better model fit compared with the exchangeable structure (Table B in S1 Appendix). The model could be expressed as:


$$\begin{array}{c} Y_{i,t,k}=\beta_{\text{0}}+\beta_{\text{1}}\;E_{i,t-m}+\sum_{j=\text{2}}^{p}\;\;\beta_{j}\;X_{i,t,j}+u_{i}+\varepsilon_{i,t} \end{array}$$ (1)

In the model, the outcome $Y_{i,t,k}$ was the cadre (k)-specific HRH density in the current year (t) for each country or territory (i). The primary exposure ($E_{i,t-m}$) was the number of epidemics occurring in prior years, with lags ranging from 0 to 5 years (m = 0, 1, …, 5, as lag 0 to lag 5 years). Previous studies show that, in addition to the immediate health impact of epidemics on the workforce, health system shocks can produce lasting workforce attrition and disruption to training pipelines [21,22]. Accordingly, we used different lags to characterize both the immediate and potential longer-term associations between epidemics and HRH density. The model also included country-level cluster effects ($u_{i}$) and was adjusted for covariates ($\;\beta_{j}\;X_{i,t,j}$), including country-year-specific urban population proportion (numerical variable), population density (numerical variable), World Bank income (categorical variable), SDI (categorical variable), and GBD super region (categorical variable). The models assumed Gaussian distributions and robust standard errors. The World Bank income and SDI groups were modeled categorically in line with previous GBD studies to align with previous studies and avoid assuming a linear association with HRH density [2,23]. The coefficient ($\beta_{\text{1}}$) was interpreted as the estimates associated with one additional epidemic on HRH density (per 10,000 population). To explore the potential heterogeneity across geographic or socioeconomic contexts, stratified analyses were conducted by GBD SDI level, World Bank income group, and GBD super region.

To quantify the burden of HRH losses attributable to epidemic exposure, we calculated two indicators, encompassing attributable person (AP) and attributable fraction (AF). AP estimates the absolute number of HRH losses that could be attributable to epidemic events in a country or territory in a given year, which could be calculated as:


$$\begin{array}{c} \begin{array}{c} {\text{AP}}_{it}=\beta_{\text{1}}\times E_{it}\times \left(\frac{P_{it}}{\text{10},\text{000}}\right) \end{array} \end{array}$$ (2)

where $\beta_{\text{1}}$ is the GEE-estimated coefficient, $E_{it}$ is the number of epidemics, and $P_{it}$ is the population counts. AF estimates the proportion of HRH losses that could be attributable to epidemics in a given country-year, expressed as:


$$\begin{array}{c} \begin{array}{c} {\text{AF}}_{it}=\frac{{\text{AP}}_{it}}{{\text{HRH}}_{it}}\times \text{10},\text{000} \end{array} \end{array}$$ (3)

which is calculated by dividing ${\text{AP}}_{it}$ by HRH count (${\text{HRH}}_{it}$) in that country-year and scaling to 10,000. AF characterizes the relative contribution of epidemics to HRH loss. This approach is conceptually analogous to the attributable fraction used in epidemiology to quantify a risk factor’s relative contribution to disease burden, extended here to indicate the proportion of HRH loss attributable to epidemics, assuming the underlying epidemic-HRH association is causal [24,25]. Comparing to association studies, this approach could provide further information on regional background exposure and health levels and temporal dynamics. Although evidence remains relatively limited, similar approaches have also been used in previous natural disaster-health studies [24,26].

We applied the Gini index and Lorenz curve to characterize the inequality of epidemics, HRHs, and epidemic-attributable HRH losses among countries and territories [27]. The Gini index quantifies the degree of inequality in a distribution, ranging from 0 (maximum equality) to 1 (maximum inequality). The Lorenz curve was constructed by plotting the cumulative share of countries against the cumulative proportion of epidemics, HRH, or epidemic-attributable HRH losses. The diagonal line represents perfect equality, and the extent to which the Lorenz curve deviates from this line reflects the degree of inequality.

To assess the robustness of our results, we conducted several sensitivity analyses. First, to examine potential reverse causation of the findings, we further investigated the association using epidemics occurring in the year after the observed HRH density as the exposure. Second, to examine epidemic intensity rather than simple event counts, we replaced the number of epidemics with the number of epidemic-related deaths. Third, to account for residual confounding related to a country’s baseline propensity for infectious diseases, we additionally adjusted for the ratio of communicable DALYs to total DALYs [18]. Fourth, to control for background long-term trends in epidemics and HRHs, we adjusted for the calendar year as a continuous linear term. Fifth, to minimize within-category residual confounding from development status, we further adjusted for SDI as a continuous variable rather than categorical. Sixth, in additional to the covariates included in the primary analysis, we adjusted for GDP per capita in the GEE models. As GDP per capita is closely correlated with SDI and World Bank income level, it was excluded from the main models to avoid multicollinearity. Seventh, instead of applying a single global epidemic-HRH association, we used region-specific *β*_1_ coefficients stratified by GBD super region, SDI level, and World Bank income group to estimate HRH losses attributable to epidemics, and compared the consistency of these results with the main model. Last, we re-estimated the association between epidemics and the proportion of each HRH cadre relative to the total HRH.

More detailed settings of the statistical analyses are provided in Texts A–C in S1 Appendix. All the GBD estimates or statistics we used in this study did not require a specific application number. All the statistical analyses were performed with R (version 4.3.1), and maps were drawn using ArcGIS desktop (version 10.1). The p values for coefficients of the GEE models were derived from Wald tests. Two-sided *p* values < 0.05 were considered statistically significant.

### Role of the funding source

The funders of this study had no role in the study design, data collection, data analysis, data interpretation, or writing of the report. All authors had full access to the data of the study and final responsibility for the decision to submit for publication.

### Artificial intelligence tools and technologies

During the preparation of this manuscript, we confirm that no artificial intelligence tools or technologies were used in the conduct of the study or in the preparation of the article content. All authors retain full responsibility for the final content and integrity of the manuscript.

## Results

### Global spatiotemporal distributions of epidemics and HRH density

**Fig 1:**
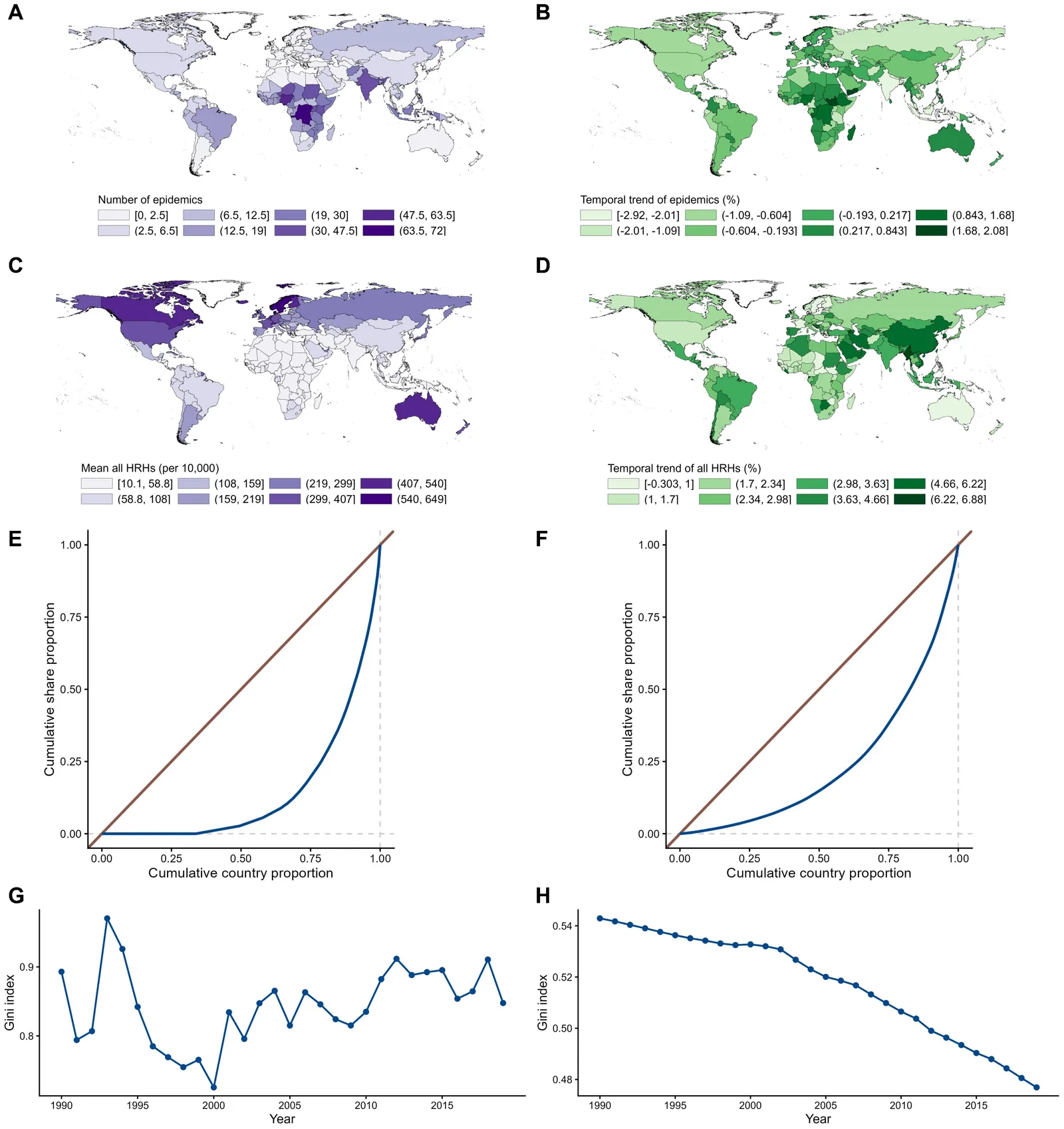
Distribution and inequality of the annual average and temporal trend of epidemics and all HRHs from 1990 to 2019. **(A)** Number of epidemics; **(B)** Temporal trend of epidemics; **(C)** Mean all HRHs (per 10,000); **(D)** Temporal trend of all HRHs; **(E)** Lorenz curve of epidemics; **(F)** Lorenz curve of all HRHs; **(G)** Gini index of epidemics; **(H)** Gini index of all HRHs. Abbreviations: HRH, human resources for health. Map shapefiles were obtained from Resources and Environmental Science Data Platform (public domain, accessible at https://www.resdc.cn/data.aspx?DATAID=205). The data are licensed for non-commercial, academic research use.

From 1990 to 2019, a total of 1,185 epidemic events were recorded worldwide, with the Democratic Republic of the Congo (72), Nigeria (55), and India (40) reporting the greatest number of epidemics (Fig 1). Meanwhile, epidemic frequency has increased over time in many low- and middle-income countries, particularly in sub-Saharan Africa, Southeast Asia, and Latin America. HRH densities were highest in high-income countries and lowest in sub-Saharan Africa and South Asia, with minimal improvement or even a decline in some sub-Saharan African countries. Fig A in S1 Appendix further demonstrates the pronounced inequality in HRH distribution and trends. High-income, Asian, and European regions showed improvements in equality over time, while widening inequality was observed in sub-Saharan Africa. Table C in S1 Appendix presents the number of epidemics and the annual average HRH density for each country from 1990 to 2019.

### Associations between epidemics and HRH density

**Fig 2:**
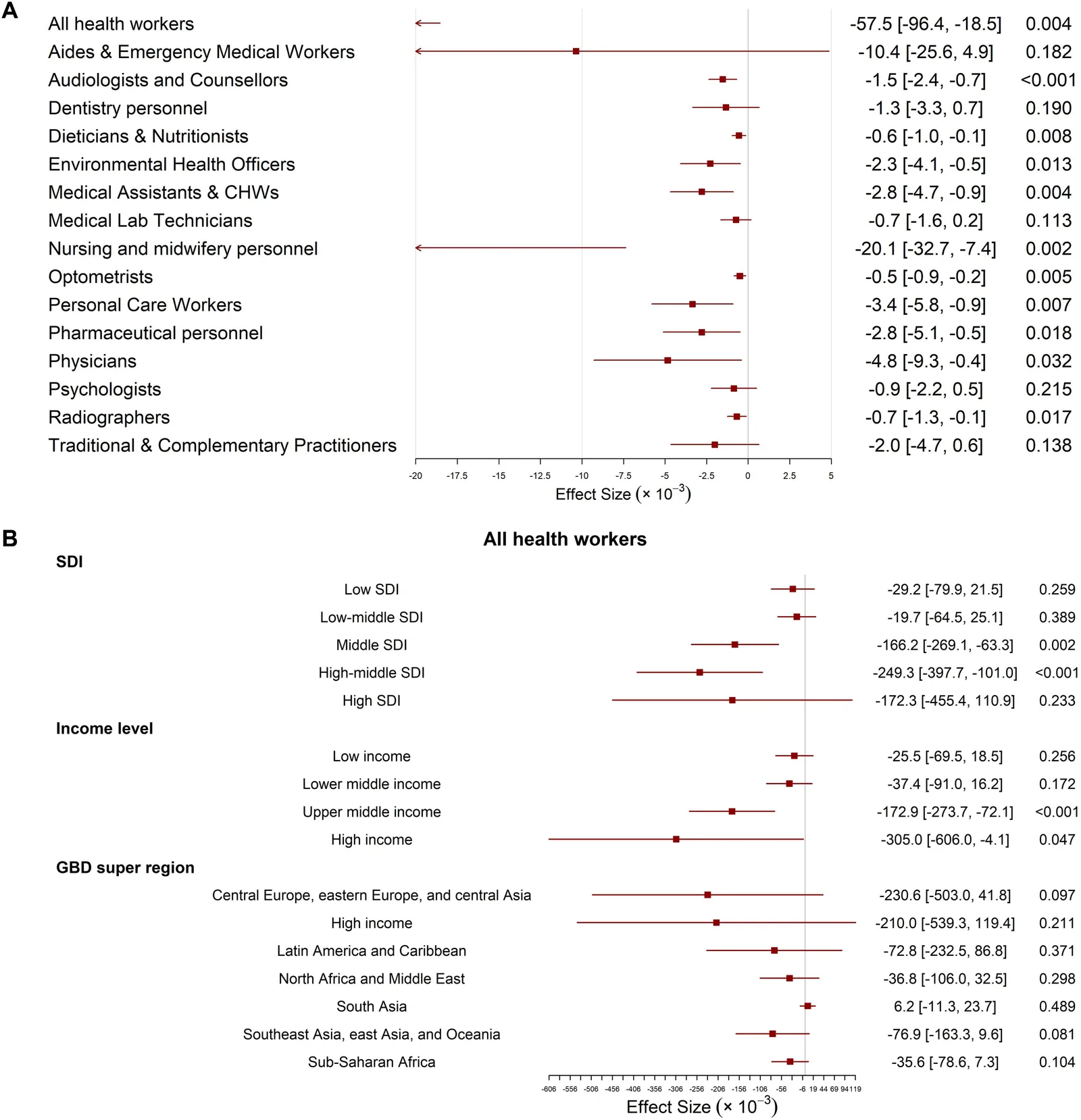
Association between epidemics and cadre-specific HRH density by SDI level, World Bank income groups, and GBD super regions. **(A)** Epidemics and cadre-specific HRHs; **(B)** Epidemics and all HRHs by SDI level, World Bank income groups, and GBD super regions. Generalized estimating equation (GEE) models were used to calculate the effect sizes. Points represent effect sizes, and error bars represent 95% confidence intervals. The *p* values for coefficients of the GEE models were derived from Wald tests. Abbreviations: CHWs, Community Health Workers; GBD, global burden of disease; HRHs, human resources for health; SDI, sociodemographic index.

The associations between epidemics and cadre-specific HRH density at different lags from 1 to 5 years are shown in Fig B in S1 Appendix. Overall, the epidemic event in the preceding year (lag 1 year) showed the strongest association. Specifically, one additional annual epidemic event in the preceding year (lag 1 year) was associated with a decrease of 57.5 × 10^−3^ (95% confidence interval (CI) [−96.4, −18.5]; *p* = 0.004) in the density of all health workers (Fig 2). Negative associations were observed across 10 HRH cadres, including Audiologists and Counsellors (*β* −1.5 × 10^−3^ (95% CI [−2.4, −0.7]; *p* < 0.001)), Dieticians and Nutritionists (*β* −0.6 × 10^−3^ (95% CI [−1.0, −0.1]; *p* = 0.008)), Environmental Health Officers (*β* −2.3 × 10^−3^ (95% CI [−4.1, −0.5]; *p* = 0.013)), Medical Assistants and CHWs (*β* −2.8 × 10^−3^ (95% CI [−4.7, −0.9]; *p* = 0.004)), Nursing and midwifery personnel (*β* −20.1 × 10^−3^ (95% CI [−32.7, −7.4]; *p* = 0.002)), Optometrists (*β* −0.5 × 10^−3^ (95% CI [−0.9, −0.2]; *p* = 0.005)), Personal Care Workers (*β* −3.4 × 10^−3^ (95% CI [−5.8, −0.9]; *p* = 0.007)), Pharmaceutical *p*ersonnel (*β* −2.8 × 10^−3^ (95% CI [−5.1, −0.5]; *p* = 0.018)), Physicians (*β* −4.8 × 10^−3^ (95% CI [−9.3, −0.4]; *p* = 0.032)), and Radiogra*p*hers (*β* −0.7 × 10^−3^ (95% CI [−1.3, −0.1]; *p* = 0.017)). Negative but non-significant associations were also found for the other cadres.

Regarding differences across socioeconomic contexts, regions with higher SDI and income levels generally showed stronger associations. For instance, one additional annual epidemic event was associated with decreases of 25.5 × 10^−3^ (95% CI [−69.5, 18.5]; *p* = 0.256), 37.4 × 10^−3^ (95% CI [−91.0, 16.2]; *p* = 0.172), 172.9 × 10^−3^ (95% CI [−273.7, −72.1]; *p* < 0.001), and 305.0 × 10^−3^ (95% CI [−606.0, −4.1]; *p* = 0.047) in the density of all health workers in low-, lower-middle-, upper-middle-, and high-income regions, respectively. Meanwhile, central Europe, eastern Europe, and central Asia and High income also showed stronger associations than other GBD super regions, but the effect estimates were statistically insignificant. Similar trends were observed for most HRH cadres, except for Traditional and Complementary Practitioners (Fig C in S1 Appendix).

### HRH losses attributable to epidemics and spatiotemporal distributions

Globally, an average of 17,549 (95% CI [5,661, 29,437]) HRH losses were attributable to epidemics annually from 1990 to 2019, corresponding to an AF of 2.57 (95% CI [0.83, 4.31]) per 10,000 HRHs (Table 1). The most affected cadres were Nursing and midwifery personnel (6,125), Aides and Emergency Medical Workers (3,162), and Physicians (1,478). Concerning AFs, the cadres with the highest affected fractions were Environmental Health Officers (5.27), Traditional and Complementary Practitioners (3.59), and Aides and Emergency Medical Workers (3.35), as shown in Table D in S1 Appendix.

**Table 1:** Global annual average HRH losses attributable to epidemics by GBD super regions, GBD regions, World Bank income groups, and SDI level from 1990 to 2019.

Group Value	Annual AP (95% CI)	Annual AF (95% CI)
Global	17,549 [5,661, 29,437]	2.57 [0.83, 4.31]
**GBD Super Region**		
Central Europe, eastern Europe, and central Asia	340 [110, 570]	0.44 [0.14, 0.74]
High income	378 [122, 634]	0.12 [0.04, 0.20]
Latin America and Caribbean	807 [260, 1,353]	1.27 [0.41, 2.13]
North Africa and Middle East	382 [123, 640]	1.77 [0.57, 2.97]
South Asia	8,758 [2,825, 14,691]	20.52 [6.62, 34.41]
Southeast Asia, East Asia, and Oceania	2,508 [809, 4,206]	1.78 [0.57, 2.98]
Sub-Saharan Africa	4,377 [1,412, 7,342]	18.03 [5.82, 30.25]
**GBD Region**		
Central Asia	26 [9, 44]	0.29 [0.09, 0.48]
Central Europe	11 [4, 19]	0.06 [0.02, 0.10]
Eastern Europe	302 [97, 506]	0.63 [0.20, 1.05]
Australasia	5 [2, 8]	0.05 [0.01, 0.08]
High-income Asia Pacific	57 [18, 96]	0.13 [0.04, 0.21]
High-income North America	242 [78, 405]	0.19 [0.06, 0.32]
Southern Latin America	18 [6, 30]	0.18 [0.06, 0.30]
Western Europe	75 [24, 125]	0.06 [0.02, 0.09]
Andean Latin America	101 [32, 169]	2.42 [0.78, 4.06]
Caribbean	33 [11, 56]	0.63 [0.20, 1.05]
Central Latin America	174 [56, 292]	0.74 [0.24, 1.25]
Tropical Latin America	482 [155, 808]	2.30 [0.74, 3.86]
North Africa and Middle East	382 [123, 640]	1.77 [0.57, 2.97]
South Asia	8,758 [2,825, 14,691]	20.52 [6.62, 34.41]
East Asia	1,018 [328, 1,708]	0.95 [0.31, 1.59]
Oceania	9 [3, 16]	3.49 [1.13, 5.86]
Southeast Asia	1,480 [477, 2,483]	4.45 [1.43, 7.46]
Central sub-Saharan Africa	908 [293, 1,523]	23.21 [7.49, 38.93]
Eastern sub-Saharan Africa	1,262 [407, 2,117]	18.68 [6.03, 31.34]
Southern sub-Saharan Africa	122 [39, 204]	2.59 [0.84, 4.35]
Western sub-Saharan Africa	2,085 [673, 3,498]	23.40 [7.55, 39.25]
**GBD SDI quintile**		
Low SDI	2,486 [802, 4,170]	19.63 [6.33, 32.93]
Low-middle SDI	11,076 [3,573, 18,579]	17.22 [5.55, 28.88]
Middle SDI	2,194 [708, 3,681]	2.38 [0.77, 3.99]
High-middle SDI	1,431 [462, 2,400]	0.69 [0.22, 1.15]
High SDI	362 [117, 607]	0.12 [0.04, 0.20]
**World Bank income level**		
Low income	3,144 [1,014, 5,274]	15.89 [5.12, 26.65]
Lower middle income	11,745 [3,789, 19,701]	12.76 [4.11, 21.40]
Upper middle income	2,269 [732, 3,806]	0.96 [0.31, 1.61]
High income	391 [126, 655]	0.12 [0.04, 0.20]

Abbreviations: AF, Attributable fraction; AP, Attributable person; GBD, global burden of disease; HRHs, human resources for health; SDI, sociodemographic index.

**Fig 3:**
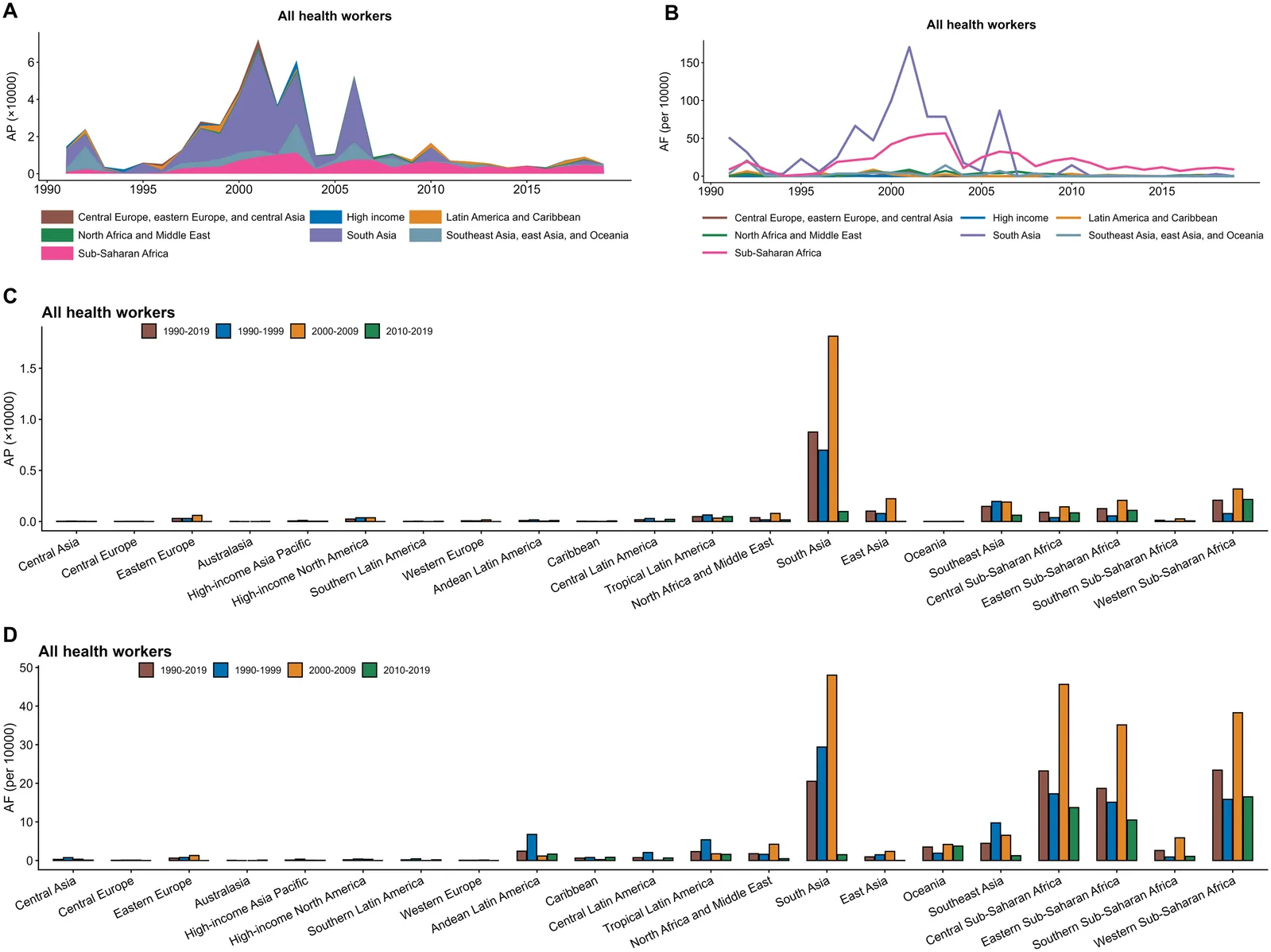
Global and regional trends of annual HRHs attributable to epidemics from 1990 to 2019, by time and GBD regions. **(A, B)** HRHs attributable to epidemics by year and GBD super regions; **(C, D)** HRHs attributable to epidemics by time period and GBD regions. Abbreviations: AF, Attributable fraction; AP, Attributable person; GBD, global burden of disease; HRHs, human resources for health; SDI, sociodemographic index.

Among GBD super regions, South Asia exhibited the greatest losses (AF 20.52; 95% CI [6.62, 34.41]), followed by sub-Saharan Africa (AF 18.03; 95% CI [5.82, 30.25]), and Southeast Asia, east Asia, and Oceania (AF 1.78; 95% CI [0.57, 2.98]) per 10,000 HRHs. Both APs and AFs were higher in 2000–2009 compared than in 1990–1999 and 2010–2019 globally and across most GBD regions (Fig 3). Among the GBD super regions, South Asia experienced the greatest HRH losses in 2000–2009 (AP 18,136; 95% CI [5,850, 30,423] and AF 48.02; 95% CI [15.49, 80.54]), followed by sub-Saharan Africa (AP 6,958; 95% CI [2,244, 11,671] and AF 31.91; 95% CI [10.29, 53.53]). GBD regions with the highest AFs during this period were Central sub-Saharan Africa (AF 45.62; 95% CI [14.72, 76.53]), Western sub-Saharan Africa (AF 38.28; 95% CI [12.35, 64.22]), and Eastern sub-Saharan Africa (AF 35.13; 95% CI [11.33, 58.93]) per 10,000 HRHs.

**Fig 4:**
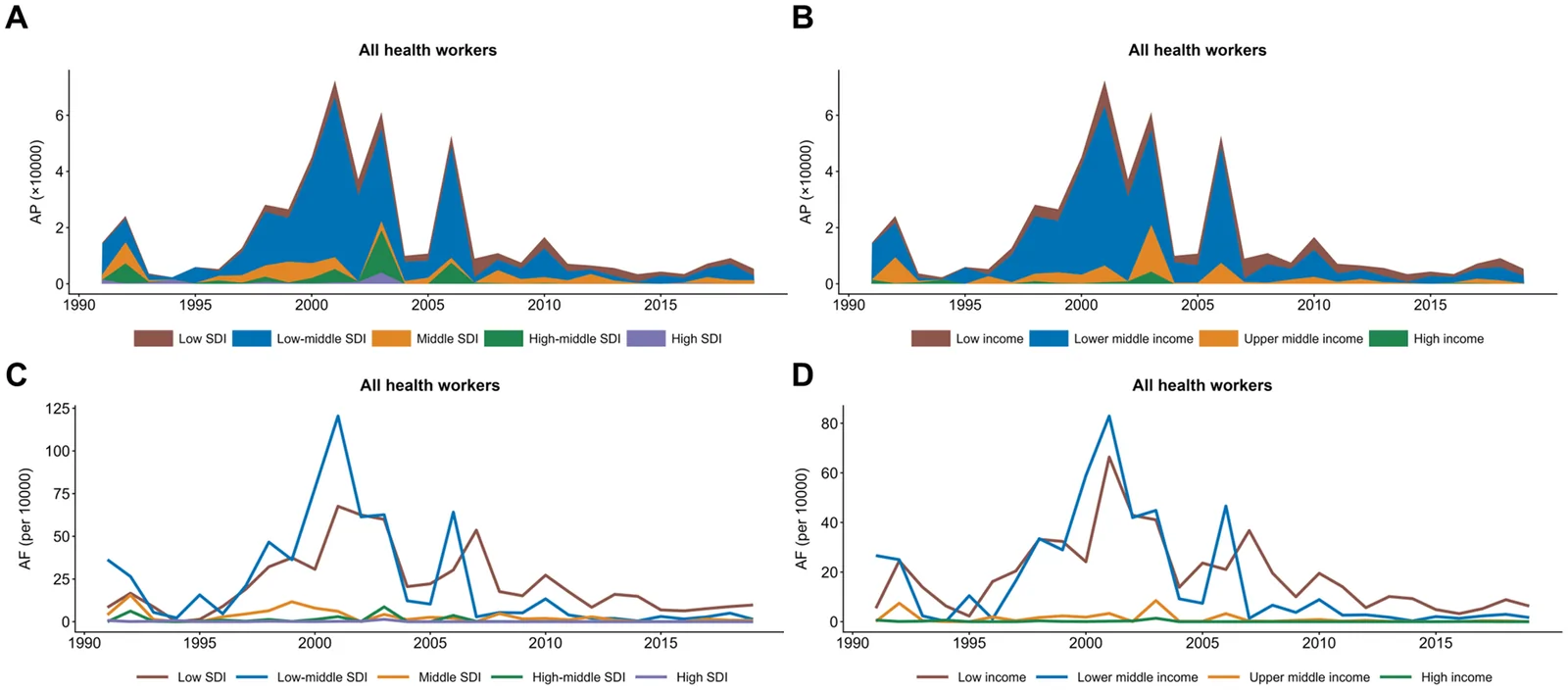
Annual AP and AF of HRHs attributable to epidemics from 1990 to 2019 by SDI and World Bank income group. **(A, B)** Annual AP by year; **(C, D)** Annual AF by year. Abbreviations: AF, Attributable fraction; AP, Attributable person; GBD, global burden of disease; HRHs, human resources for health; SDI, sociodemographic index.

Concerning socioeconomic contexts, our analysis revealed a clear socioeconomic gradient in HRH losses, with higher AFs observed in regions with lower SDI and income levels (Fig 4). The AFs were 19.63 (95% CI [6.33, 32.93]), 17.22 (95% CI [5.55, 28.88]), 2.38 (95% CI [0.77, 3.99]), 0.69 (95% CI [0.22, 1.15]), and 0.12 (95% CI [0.04, 0.20]) per 10,000 HRHs in low-, low-middle-, middle-, high-middle, and high-SDI level regions, and 15.89 (95% CI [5.12, 26.65]), 12.76 (95% CI [4.11, 21.40]), 0.96 (95% CI [0.31, 1.61]), 0.12 (95% CI [0.04, 0.20]) per 10,000 HRHs in low-, lower-middle-, upper-middle-, and high-income level regions. HRH losses attributable to epidemics, especially in regions with lower socioeconomic settings, were also concentrated in 2000–2009. Specifically, the annual AFs were 17.82 (95% CI [5.75, 29.89]), 28.41 (95% CI [9.16, 47.66]), and 8.23 (95% CI [2.65, 13.80]) for low-income countries, and 16.38 (95% CI [5.28, 27.47]), 27.30 (95% CI [8.80, 45.79]), and 2.54 (95% CI [0.82, 4.26]) per 10,000 HRHs for low-middle-income countries in 1990–1999, 2000–2009, and 2010–2019, respectively.

**Fig 5:**
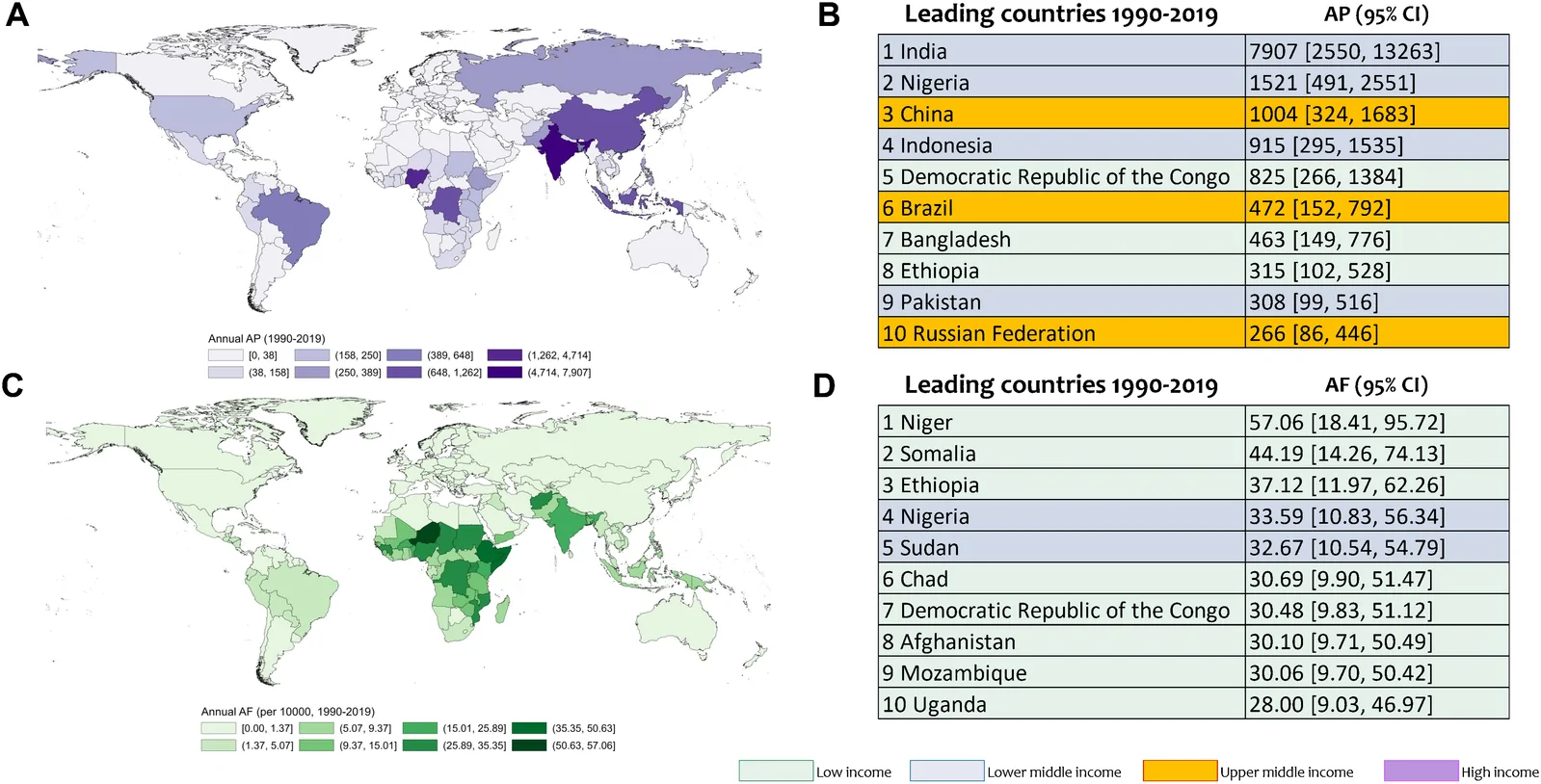
World map and leading 10 countries with greatest annual AP and AF of HRHs attributable to epidemics from 1990 to 2019. **(A)** Country-level AP; **(B)** Leading 10 countries for AP; **(C)** Country-level AF; **(D)** Leading 10 countries for AF. Abbreviations: AF, Attributable fraction; AP, Attributable person; HRHs, human resources for health; SDI, sociodemographic index. Map shapefiles were obtained from Resources and Environmental Science Data Platform (public domain, accessible at https://www.resdc.cn/data.aspx?DATAID=205). The data are licensed for non-commercial, academic research use.

Fig 5 shows the country-specific annual APs and AFs from 1990 to 2019 (detailed estimates are available in Table E in S1 Appendix), highlighting the leading 10 countries. The greatest AP values were found in South Asia and sub-Saharan Africa, with India (AP 7,907; 95% CI [2,550, 13,263]), Nigeria (AP 1,521; 95% CI [491, 2,551]), and China (AP 1,004; 95% CI [324, 1,683]) showing the greatest estimates. The highest AFs were observed primarily in sub-Saharan Africa, with Niger (AF 57.06; 95% CI [18.41, 95.72]), Somalia (AF 44.19; 95% CI [14.26, 74.13]), and Ethiopia (AF 37.12; 95% CI [11.97, 62.26]) per 10,000 HRHs showing the greatest values.

### Sensitivity analyses

Our sensitivity analyses demonstrated the overall robustness of the primary findings against alternative explanations and potential unmeasured confounders. When applying future epidemic events after the year of HRH data as the exposure, we observed no significant association with historical HRH (Fig D in S1 Appendix). Using epidemic-related deaths to represent event intensity yielded significant associations with health workforce declines generally in line with the primary epidemic count-based exposure (Fig E in S1 Appendix). Adjusting for baseline disease propensity using the ratio of communicable DALYs to total DALYs yielded consistent negative associations across major cadres (Fig F in S1 Appendix). When adding calendar year to account for long-term secular trends (Fig G in S1 Appendix) or adjusting for SDI continuously rather than categorically (Fig H in S1 Appendix), the associations between epidemics and HRH remained robust. Models additionally included GDP per capita as a covariate generally yielded similar estimates, although some estimates were attenuated (Fig I in S1 Appendix). Using region-specific estimates stratified by SDI levels and World Bank income groups, the global annual AFs from 1990 to 2019 were 2.74 (95% CI [−0.23, 5.70]) and 2.62 (95% CI [−0.21, 5.46]) per 10,000 HRHs, respectively (Table F in S1 Appendix), which were comparable to the main results using the single global estimate (AF 2.57; 95% CI [0.83, 4.31]). Country-specific estimates also demonstrated relatively higher consistency when using region-specific estimates stratified by SDI levels, World Bank income groups, and GBD super regions, with determination coefficients (*R*^2^) of 0.766, 0.800, and 0.888, respectively (Fig J in S1 Appendix). In addition, we found epidemics were associated with decreased proportions of Dieticians and Nutritionists, Optometrists, Personal Care Workers, and Pharmaceutical personnel, as well as increased proportions of Environmental Health Officers (Fig K in S1 Appendix).

### Discussion

To the best of our knowledge, this is the most up-to-date comprehensive study of the global, regional, and national HRH losses attributable to epidemics. We found that epidemics were associated with a decrease in the density of all HRHs and nearly all cadre-specific HRHs. Assuming the observed epidemic-HRH relationships were causal, globally, we estimated substantial HRH losses of approximately 17.5 thousand each year (representing approximately 2.6 per 10,000 HRHs) attributable to epidemics from 1990 to 2019, with a pronounced estimate from 2000 to 2009. South Asia and sub-Saharan Africa showed the highest AFs. Meanwhile, concerning socioeconomic context, low- and lower-middle-income countries showed higher AFs. The top 10 countries with the highest AFs were observed primarily in sub-Saharan Africa, such as Niger, Somalia, and Ethiopia.

Overall, our analyses suggest that epidemics are significantly associated with decreases in healthcare workforce. The associations were stronger in the 1-year lag window compared to other different lagged windows. Following an epidemic disaster event, the healthcare delivery system may experience an immediate surge in service demand compounded by heightened occupational exposure among frontline personnel [28]. This immediate pressure may frequently bring severe personnel shortages, overwhelming workloads, and widespread professional burnout, thereby disrupting the operational equilibrium of the health infrastructure [28,29]. Notably, health systems in low-resource settings are always accompanied by limited systemic resilience while bearing a disproportionate burden of these shocks [30]. Consequently, these low-resource regions possess a lower capacity to absorb such disruptions, which may be associated with more pronounced and severe losses that enlarge the persistent HRH inequality across geographic and socioeconomic strata. This inequality across geographic and socioeconomic strata may be a major barrier to progress toward SDG target 3.C for equitable HRH distribution [8]. The compounded burden of epidemics on HRH losses poses a significant threat to population health outcomes in vulnerable regions worldwide. Without immediate and targeted policy interventions to strengthen HRH development, future disaster events could be associated with even more severe and lasting damage [31,32]. These effects would extend beyond the loss of HRH, destabilizing essential health services across entire health systems. Such disruptions are particularly concerning in areas already facing critical shortages of certain healthcare professionals, especially nurses and midwives, physicians, and pharmaceutical personnel [2].

While previous research has focused on a more limited category of healthcare workforce, concentrating on doctors and nurses, the following drivers, including remuneration and security problems career prospects, working environment, and job satisfaction were the major factors associated with health workers’ migration or intention to migrate from vulnerable regions over the past half-century [33]. However, the occurrence of some extreme situations, such as the COVID-19 pandemic, may also present unexpected opportunity to increase innovation in the interactions and development between higher education systems and healthcare systems globally [34,35]. Our analysis demonstrates that epidemics occurring globally over the past three decades are associated with profound and sustained reductions of HRH density, with immediate and delayed impacts disproportionately affecting critical roles, notably midwifery personnel, physicians, and personal care workers.

Beyond cadres with direct clinical exposure to infectious disease, we also observed reduced density among cadres less directly involved in outbreak response, including Dieticians and Nutritionists, Environmental Health Officers, Optometrists, and Radiographers. A plausible explanation is the reallocation of these workers toward epidemic response and case management during outbreaks, particularly in countries with limited HRH capacity where staff are commonly redeployed across cadres out of necessity [36]. Disruption to routine, non-emergency health services during epidemics may also reduce demand for these cadres specifically. More broadly, because the HRH density measure captures the actively employed workforce identified through labor-force surveys, censuses, and National Health Workforce Accounts, an observed decline could reflect several non-mutually-exclusive pathways beyond direct mortality and morbidity, including temporary or permanent redeployment within the health sector or international migration [30,37]. However, our ecological design could not distinguish between these pathways, and thus further individual-based prospective studies are warranted to examine these hypotheses. These disproportionate losses increase pre-existing HRH shortages, disrupt the skill-mix balance, and worsen the geographic maldistribution. Concerted global action is urgently needed to build resilience among these critical HRH categories through targeted interventions and improve preparedness for future health emergencies.

Our analysis also shows that between 2000 and 2009, South Asia and sub-Saharan Africa experienced especially severe HRH losses attributable to epidemics. These losses occurred during high-burden epidemics that revealed underlying weaknesses in the health system. According to EM-DAT records, key epidemics associated with these declines included: in South Asia, cholera (i.e., Bangladesh, 2007), acute watery diarrhea of unknown etiology (i.e., Bangladesh, 2000), Chikungunya virus disease (i.e., India, 2005–2006); in sub-Saharan Africa, cholera (i.e., Malawi, 2001), and influenza (i.e., Democratic Republic of the Congo, 2002–2003), Meningococcal disease (i.e., Nigeria, 2009), Chikungunya (i.e., Réunion, 2005), and acute watery diarrhea of unknown etiology (i.e., Ethiopia, 2006). Importantly, the threat of some pathogens, such as chikungunya and dengue, is circulating between Asia and Africa and expanding their reach as greater threats [38,39]. Inadequate surveillance capacity has accelerated the geographic distribution of novel genotypes, and recurrent outbreaks have further weakened the resilience of the health systems in these regions. Given that some of these pathogens are highly susceptible to waterborne transmission, the establishment of national or regional wastewater or environmental laboratories for multi-pathogen surveillance might help reduce disease incidence and alleviate the burden of repetitive tasks on healthcare workers [40,41]. Consequently, medical professionals could redirect their efforts toward skill development and capacity building, ultimately enhancing the healthcare systems. Meanwhile, vaccines against gastroenteritis have gained significant progress these days, promotion of vaccine coverage or appropriate medications for vulnerable populations could lead to further savings of HRH in these regions [42–44]. The international community needs to prioritize coordinated technological, financial, and educational support to reduce epidemic-attributable disease burden and safeguard HRH sustainability [45].

A key strength of this study lies in its comprehensive quantification of epidemic-attributable HRH losses at the global, regional, and national levels over three decades. By capturing both immediate and delayed estimates across all workforce cadres, our analysis provides novel evidence that advances beyond prior research, which has focused predominantly on high-income settings or only on overall HRH density.

Our study had several limitations. First, while EM-DAT served as the primary data source for identifying and assessing multi-hazard incidents in this study, it could not record all hazard events, especially in vulnerable regions [46]. Meanwhile, changes in geography also impact the reporting pattern of disaster events across different spaces and times. These could all lead to underreporting in certain regions and, therefore, cause underestimated inequalities in vulnerable areas. Second, while our analysis spans three decades (1990–2019), it did not capture the influences of the global crisis of the COVID-19 pandemic due to limited cadre-specific HRH data after 2019. Although this omission precludes insights into the most recent pandemic, the near-simultaneous global saturation of COVID-19 obscured the geographic inequalities in workforce vulnerability that are central to our analysis. Future research should compare these findings with COVID-19-attributable workforce losses to identify novel epidemic-related attrition pathways for healthcare workers. Third, because EM-DAT relies on various reporting sources, including national authorities and the media, differences in surveillance systems and reporting capacities across countries may affect the completeness of event counts. Countries with stronger reporting infrastructure may appear to experience more events than those with limited capacity. This variation may influence comparisons across regions or income groups and should be considered when interpreting these results. Fourth, our study established a single global epidemic-HRH association based on all available evidence, in line with typical health impact assessment methods, which may fail to characterize epidemic-HRH associations across different geographic and socioeconomic settings. Nevertheless, we repeated the analyses based on region-specific associations stratified by SDI groups, World Bank income levels, and GBD super regions, and the estimates also showed high consistency with the main results. Further studies based on finer geographic data are warranted to improve the accuracy of dose–response associations and projected estimates. Finally, our model did not account for the international migration of HRH, which is a major confounding driver of health workforce inequality [33]. Epidemics may accelerate the migration of healthcare professionals from resource-limited regions to high-resource ones, potentially confounding the estimates in our analysis. Future research should integrate transnational migration flow data to better disentangle epidemic-induced workforce losses from broader global mobility patterns. Generally, while our analysis identified broad trends in HRH attrition, the mechanisms driving this phenomenon warrant further investigation. The global-scale nature of our panel data provides a valuable macro-level perspective on structural vulnerability, yet it underscores the need for context-specific, longitudinal research to inform targeted local workforce policies.

In conclusion, epidemics were associated with substantial and sustained HRH losses (approximately 17.5 thousand annually, representing approximately 2.6 per 10,000 HRHs), with notable geographic and socioeconomic inequalities in the last three decades. South Asia and sub-Saharan Africa exhibited the highest epidemic-associated HRH losses. Regarding socioeconomic contexts, low- and lower-middle-income countries experienced a higher burden. Epidemic preparedness and response should prioritize context- and cadre-specific workforce protection and capacity, especially in lower-resource settings and for the cadres most affected, in order to mitigate workforce vulnerabilities and promote global HRH equity.

## Supporting information

S1 Checklist Reporting of Studies Conducted using Observational Routinely-Collected Data (RECORD) guideline. (available from https://www.record-statement.org).
(PDF)

S2 Checklist Guidelines for Accurate and Transparent Health Estimates Reporting (GATHER) statement. The GATHER checklist is adapted from Stevens GA, Alkema L, Black RE, Boerma JT, Collins GS, Ezzati M, et al. Guidelines for accurate and transparent health estimates reporting: The GATHER statement. PLOS Med.2016. doi: http://dx.doi.org/10.1371/journal.pmed.1002056. (available from https://www.equator-network.org/reporting-guidelines/gather-statement).
(PDF)

S1 Appendix Supplementary information. **Text A.** Data collection. **Text B.** Longitudinal modeling. **Text C.** Global modeling of epidemic-attributable health workforce impact. **Table A.** Overview of Data Sources and Variables. **Table B.** Quasi-likelihood under the Independence model Criterion (QIC) comparison for Generalized Estimating Equation correlation structures. **Table C.** Details on the number of epidemics and the annual average HRH density for each country (1990–2019). **Fig A.** Region-specific Lorenz curves and Gini indices for epidemics and all HRHs. **Fig B.** Association between epidemics and cadre-specific HRHs at different lags (0–5 year). **Fig C.** Association between epidemics and cadre-specific HRH density by SDI level, World Bank income groups, and GBD super regions. **Table D.** Global annual average cadre-specific HRH losses attributable to epidemics from 1990 to 2019 among all countries and territories. **Table E.** Annual average HRH losses attributable to epidemics from 1990 to 2019 among countries and territories. **Fig D.** Association between epidemics occurring after the HRH observation year and HRH density. **Fig E.** Association between epidemic intensity (measured by epidemic-related deaths) and HRH density. **Fig F.** Association between epidemics and HRH density adjusting for communicable DALYs ratio. **Fig G.** Association between epidemics and HRH density additionally adjusting for calendar year. **Fig H.** Association between epidemics and HRH density using continuous SDI adjustment. **Fig I.** Association between epidemics and density of HRH cadres in the sensitivity analyses additionally adjusting GDP per capita. **Table F.** Global annual average HRH losses attributable to epidemics using the estimates of GBD super regions, SDI level, and World Bank income groups. **Fig J.** Consistency of annal AF value between main model (global) and estimates by SDI level, World Bank income groups, and GBD super regions. **Fig K.** Association between epidemics and the proportion of each HRH cadre.
(DOCX)

## Abbreviations

AF: attributable fraction
AP: attributable person
CHWs: Community Health Workers
CI: confidence interval
COVID-19: coronavirus disease 2019
DALYs: disability-adjusted life-years
EM-DAT: Emergency Events Database
GATHER: Guidelines for Accurate and Transparent Health Estimates Reporting
GBD: Global Burden of Disease
GDP: gross domestic product
GEE: generalized estimating equation
HRH: human resources for health
ISCO: International Standard Classification of Occupations
MERS: Middle East Respiratory Syndrome
NHWA: National Health Workforce Accounts
QIC: quasi-likelihood information criterion
RECORD: Reporting of Studies Conducted using Observational Routinely-Collected Data
SDI: Socio-demographic Index
WHO: World Health Organization
