# Supplementary material for: Global, regional, and national impact of epidemic disasters on health workforce equality between 1990 and 2019: An ecological and modeling study
Source: PLoS Med. 2026 Sep 22;23(9):e1005134. doi: 10.1371/journal.pmed.1005134 (PMC13596819; doi:10.1371/journal.pmed.1005134)
Supplement: Supplementary file 3 [file pmed.1005134.s003.docx]

**S1 Appendix. Supplementary Information**

Table of Contents

[Text A. Data collection	2](#_Toc237185204)

[Text B. Longitudinal modeling	4](#_Toc237185205)

[Text C. Global modeling of epidemic-attributable health workforce impact	6](#_Toc237185206)

[Table A. Overview of Data Sources and Variables	7](#_Toc237185207)

[Table B. Quasi-likelihood under the Independence model Criterion (QIC) comparison for Generalized Estimating Equation correlation structures	8](#_Toc237185208)

[Table C. Details on the number of epidemics and the annual average HRH density for each country (1990–2019)	9](#_Toc237185209)

[Fig A. Region-specific Lorenz curves and Gini indices for epidemics and all HRHs.	15](#_Toc237185210)

[Fig B. Association between epidemics and cadre-specific HRHs at different lags (0-5 year).	18](#_Toc237185211)

[Fig C. Association between epidemics and cadre-specific HRH density by SDI level, World Bank income groups, and GBD super regions.	29](#_Toc237185212)

[Table D. Global annual average cadre-specific HRH losses attributable to epidemics from 1990 to 2019 among all countries and territories.	30](#_Toc237185213)

[Table E. Annual average HRH losses attributable to epidemics from 1990 to 2019 among countries and territories	31](#_Toc237185214)

[Fig D. Association between epidemics occurring after the HRH observation year and HRH density.	36](#_Toc237185215)

[Fig E. Association between epidemic intensity (measured by epidemic-related deaths) and HRH density.	37](#_Toc237185216)

[Fig F. Association between epidemics and HRH density adjusting for communicable DALYs ratio.	38](#_Toc237185217)

[Fig G. Association between epidemics and HRH density additionally adjusting for calendar year.	39](#_Toc237185218)

[Fig H. Association between epidemics and HRH density using continuous SDI adjustment.	40](#_Toc237185219)

[Fig I. Association between epidemics and density of HRH cadres in the sensitivity analyses additionally adjusting GDP per capita.	41](#_Toc237185220)

[Table F. Global annual average HRH losses attributable to epidemics using the estimates of GBD super regions, SDI level, and World Bank income groups.	42](#_Toc237185221)

[Fig J. Consistency of annal AF value between main model (global) and estimates by SDI level, World Bank income groups, and GBD super regions.	43](#_Toc237185222)

[Fig K. Association between epidemics and the proportion of each HRH cadre.	44](#_Toc237185223)

## Text A. Data collection

**Epidemic Data**

Epidemic event data were extracted from the Emergency Events Database (EM-DAT), a widely recognized global disaster database maintained by the Centre for Research on the Epidemiology of Disasters (CRED) at the Université catholique de Louvain, Belgium. Established in 1988, EM-DAT collects information on both natural and technological disasters worldwide. Its data are compiled from multiple sources, including United Nations agencies, non-governmental organizations, reinsurance companies, research institutes, and media reports.

EM-DAT includes only major disaster events, based on specific inclusion criteria at the country level: (a) at least 10 reported deaths, (b) 100 or more individuals affected, (c) a declared state of emergency, (d) or an official call for international assistance. For this study, we calculated the annual number of epidemic events per country or territory based on EM-DAT records. A total of 1185 epidemic records occurring in 132 countries and territories from 1990-2019 were included in our final analyses.

**Human Resources for Health (HRH) data**

The data on human resources for health (HRH) were derived from a systematic analysis for the Global Burden of Disease Study 2019 (GBD 2019). This comprehensive dataset spans from 1990 to 2019 and includes annual data on health workforce densities per 10,000 population for various health worker cadres across 204 countries and territories consistent with the GBD study. The data was primarily identified through the International Labour Organization and Global Health Data Exchange databases, which provided 1404 country-years of data from labour force surveys and 69 country-years of census data with detailed microdata on health-related employment. Additionally, 2950 country-years of data were obtained from the WHO National Health Workforce Accounts (NHWA).

All occupational responses were mapped to the International Standard Classification of Occupations 1988 (ISCO‑88) three‑ or four‑digit codes. After harmonizing source‑specific coding, 16 HRH cadres were defined and estimated. To correct for inconsistencies in case definitions, reporting standards, and double‑counting, the GBD study also applied a lasso regression–derived crosswalk approach. Matched pairs of NHWA versus survey/census observations informed location‑ and region‑specific adjustment factors, regularized via Gaussian priors to mitigate overfitting where matched data were sparse. All cadre‑specific results were internally rescaled to align with total HRH and overall employment counts, ensuring that the sum of component cadres equaled the total health workforce estimate for each country‑year.

In our study, we finally included a total of 16 HRH cadres: All health workers (total HRH), Aides & Emergency Medical Workers, Audiologists and Counsellors, Dentistry personnel, Dieticians & Nutritionists, Environmental Health Officers, Medical Assistants & Community Health Workers (CHWs), Medical Lab Technicians, Nursing and midwifery personnel, Optometrists, Personal Care Workers, Pharmaceutical personnel, Physicians (Doctors), Psychologists, Radiographers, and Traditional & Complementary Practitioners.

**Covariates**

A set of socioeconomic and demographic covariates was included to control for potential confounding. All covariates were selected from internationally recognized databases to ensure consistency in definitions and comparability across countries. The covariates were primarily sourced from the GBD study, the World Bank database (https://www.worldbank.org/), and Our World in Data (https://ourworldindata.org/), which compiles data from the United Nations Development Programme (UNDP).

The proportion of the population living in urban areas, expressed as a percentage, was sourced from the World Bank. This variable is based on national definitions of “urban”, which vary by country, and is calculated using World Bank population estimates and urbanization ratios from the United Nations Population Division. Population density from the World Bank, was calculated as the midyear population divided by land area in square kilometers. Population estimates follow a de facto approach, including all residents regardless of citizenship or legal status, but excluding refugees not permanently settled in the country of asylum. Countries were classified into four income groups (low, lower-middle, upper-middle, and high income) according to the World Bank’s annual income-level classification. Meanwhile, the Socio-demographic Index (SDI), derived from the Global Burden of Disease (GBD) study, was also included. SDI is a composite indicator combining lag-distributed income per capita, average educational attainment (for those aged ≥15 years), and total fertility rate among females under 25 years of age. The index is scaled between 0 (least developed) and 1 (most developed). SDI values were grouped into five levels (low, low-middle, middle, high-middle, and high) in accordance with the GBD study. Geographic classifications were based on GBD 21 regions and seven super-regions. GBD regions consist of countries that are geographically and epidemiologically similar, and these are further grouped into seven super-regions: (a) Central Europe, eastern Europe, and central Asia; (b) High income; (c) Latin America and Caribbean; (d) North Africa and Middle East; (e) South Asia; (f) Southeast Asia, east Asia, and Oceania; and (g) Sub-Saharan Africa. Several covariates were considered in our sensitivity analyses for their potential overlaps with aforementioned covariates. The gross domestic product (GDP) per capita was obtained from the World Bank database. GDP per capita is formally defined as the gross domestic product divided by the midyear population, used as a measure of averaged gross value within an economy.

## Text B. Longitudinal modeling

**Panel data construction**

The dataset was organized into a country-year panel covering the years 1990 to 2019. Each observation represents a specific country in a given year, allowing for both cross-country and within-country variation over time. The primary outcome variable was the density of each HRH cadre, and the exposure variable was the annual number of epidemic events per country. To control for potential confounding, urban population proportion (numerical variable), population density (numerical variable), world bank income (categorical variable), SDI (categorical variable) and GBD super region (categorical variable) were included as the covariates. Missing values in the covariates were addressed using linear interpolation. Linear interpolation is a practical and commonly used method in time-series and panel data analysis, particularly when the data are assumed to change gradually over time. By filling internal gaps, this method preserves the panel structure and maximizes the number of observations available for modeling. The final dataset included 194 countries or territories that had available data in the panel.

**Descriptive statistics and trend analysis**

Descriptive statistics were first conducted to summarize the total number of epidemic events and the average HRH density for each country over the study period (1990–2019).

Temporal trends in epidemic events and HRH density were evaluated using log-linear regression models to estimate the annual percent change. This approach assumes that the outcome changes at a constant percentage rate each year, corresponding to a linear trend on a logarithmic scale. The regression model is expressed as:

$$\begin{aligned} log(R_{y})=\beta_{0}+\beta_{1}y\#(1) \end{aligned}$$

where $R_{y}$ is the value of the variable (either epidemic count or HRH density) in year $y$, $\beta_{0}$ is the intercept, and $\beta_{1}$ is the slope representing the average yearly change on the log scale.

The annual percent change is then calculated using the estimated slope ($\beta_{1}$) as:

$$\begin{aligned} \text{Annual percent change}=\left( e^{\beta_{1}}-1 \right)\times100\%\#(2) \end{aligned}$$

To assess the cross-country inequality in the distribution of epidemic events and HRH density across countries and territories, the Gini index and Lorenz curve were used. The Gini index quantifies the degree of inequality in a distribution, ranging from 0 (maximum equality) to 1 (maximum inequality).

The Lorenz curve was constructed by plotting the cumulative share of countries, ranked in ascending order of the variable, against the cumulative proportion of the total value. The diagonal line represents absolute equality, and the extent to which the Lorenz curve deviates from this line reflects the degree of inequality.

**Generalized Estimating Equation (GEE) model**

To examine the longitudinal association between epidemic events and HRH while accounting for time-varying covariates and within-country correlations, a Generalized Estimating Equation (GEE) model was applied. The GEE approach extends the generalized linear model (GLM) framework to account for correlated data, which is common in longitudinal settings where repeated observations are made for the same units over time. In this study, countries are observed annually from 1990 to 2019, resulting in a country-year panel with inherent within-country correlation. The GEE model estimates population-averaged effects, which represent the average association across all countries.

The GEE model specification was as follows:

$$\begin{aligned} Y_{i,t,k}=\beta_{0}+\beta_{1} E_{i,t-m}+\sum_{j=2}^{p} \beta_{j} X_{i,t,j}+u_{i}+\varepsilon_{i,t}\#(4) \end{aligned}$$

where the outcome variable was the cadre ($k$)-specific HRH density in the current year ($t$) of one country or territory ($i$). The primary exposure variable ($E_{i,t-m}$) was the number of epidemics in the prior $m$ ($m$ = 0, 1, …, 5, as Lag 0 to Lag 5 year) year, $u_{i}$ represents the country-level cluster effect and $\varepsilon_{i,t}$ is the within-country error term. The covariates ($X_{i,t,j}$) included urban population proportion (numerical variable), population density (numerical variable), world bank income (categorical variable), SDI (categorical variable) and GBD super region (categorical variable). To address temporal autocorrelation, we employed an autoregressive correlation structure of order 1 (AR1). All models assumed a Gaussian distribution. Parameter estimates were obtained using robust variance estimators. The coefficient $\beta_{1}$ was interpreted as the effect of one additional epidemic on the HRH density (per 10,000 population).

To explore potential effect heterogeneity, stratified analyses were conducted. Separate GEE models were fitted within subgroups defined by GBD SDI level, World Bank income group, and GBD super region. This allowed for the assessment of whether the association between epidemic frequency and HRH density differs by level of development or geographic context.

## Text C. Global modeling of epidemic-attributable health workforce impact

**Attributable Measures**

To quantify the global impact of epidemics on health workforce availability, we projected epidemic-attributable measures, which were derived by combining the global effect estimate from the GEE model with country-level data on population, epidemic frequency, and HRH density.

Attributable Persons (APs) represented the number of HRH personnel could be affected by epidemic events of a country or territory in a given year. The number of attributable HRH is calculated as:

$$\begin{aligned} \text{AP}_{it}=\beta_{1}\times E_{it}\times\left( \frac{P_{it}}{10,000} \right)\#\left( 5 \right) \end{aligned}$$

where $\beta_{1}$ is the GEE-estimated coefficient, $E_{it}$ is the number of epidemics, and $P_{it}$ is the population in country $i$, year $t$. This yields the number of HRH lost or affected due to epidemic exposure in absolute terms.

The Attributable Fraction (AF) estimated the proportion of total HRH density that could be affected by epidemics in a given country/territory-year. This measure reflects the relative burden of epidemics on HRH, calculated by dividing APs by the total HRH count in that country-year and scaling to per 10,000:

$$\begin{aligned} \text{AF}_{it}=\frac{\text{AP}_{it}}{\text{HRH}_{it}}\times10,000\#\left( 6 \right) \end{aligned}$$

**Inequality in epidemic-attributable health workforce impact**

To assess how the HRH burden attributable to epidemics is distributed across countries, inequality analyses were conducted using the Gini index and Lorenz curve. The Gini index quantifies inequality, with values closer to 1 indicating greater disparity. Lorenz curves provide a visual depiction by plotting the cumulative share of countries against the cumulative proportion of epidemic-attributable HRH impact.

## Table A. Overview of Data Sources and Variables

| Variable Name | Source Database | Definition/Description | Units/Scale |
| --- | --- | --- | --- |
| Epidemics | EM-DAT | Count of major epidemic events meeting EM-DAT criteria (≥10 fatalities, ≥100 affected, state of emergency, or call for international assistance) | Count |
| Human Resources for Health (HRH) Density | GBD Study | Density of different HRH types (e.g., physicians, nurses) | Per 10,000 population (assumed based on typical HRH metrics) |
| Urban Population Proportion | World Bank | Percentage of people living in urban areas as defined by national statistical offices | % |
| Population Density | World Bank | Midyear population divided by land area | People per sq. km |
| World Bank Income | World Bank | Categorization of countries into Low, Lower-Middle, Upper-Middle, and High income groups | Categorical (4 groups) |
| Socio-Demographic Index (SDI) | GBD Study | Composite measure of development (income, education, fertility), into low, low-middle, middle, high-middle, and high levels | Categorical (5 groups) |
| GBD Super Regions and Regions | GBD Study | Geographical classifications based on epidemiological similarity and cause-of-death patterns | Categorical (7 super regions, 21 regions) |
| GDP per capita | World Bank | Gross Domestic Product divided by midyear population | Current US$ |
| Communicable and total disability-adjusted life-years (DALYs) burden | GBD Study | The ratio of communicable DALYs to total DALYs | % |

Abbreviations: DALYs, disability-adjusted life-years; EMDAT, Emergency Events Database; GBD, global burden of disease; HRHs, human resources for health; SDI, sociodemographic index.

## Table B. Quasi-likelihood under the Independence model Criterion (QIC) comparison for Generalized Estimating Equation correlation structures

| **Outcome** | **AR1** | **Exchangeable** |
| --- | --- | --- |
| All health workers | 2455.2 | 3765.2 |
| Aides & Emergency Medical Workers | 591.6 | 671.9 |
| Audiologists and Counsellors | 3.2 | 4.1 |
| Dentistry personnel | 7.1 | 8.2 |
| Dieticians & Nutritionists | 1.5 | 2.1 |
| Environmental Health Officers | 3.1 | 3.2 |
| Medical Assistants & CHWs | 15.7 | 17.0 |
| Medical Lab Technicians | 6.8 | 8.6 |
| Nursing and midwifery personnel | 262.5 | 343.1 |
| Optometrists | 0.7 | 0.9 |
| Personal Care Workers | 37.9 | 50.7 |
| Pharmaceutical personnel | 8.5 | 12.4 |
| Physicians | 30.6 | 36.1 |
| Psychologists | 4.8 | 5.5 |
| Radiographers | 1.8 | 3.0 |
| Traditional & Complementary Practitioners | 1.8 | 1.9 |

Abbreviations: CHWs, Community Health Workers.

## Table C. Details on the number of epidemics and the annual average HRH density for each country (1990–2019)

| **GBD Super Region** | **GBD Region** | **Country name** | **GBD SDI Quintile** | **World Bank income level** | **Number of epidemics** | **Average of HRH density** |
| --- | --- | --- | --- | --- | --- | --- |
| Central Europe, eastern Europe, and central Asia | Central Asia | Armenia | Middle SDI | Lower middle income | 0 | 109.87 |
| Central Europe, eastern Europe, and central Asia | Central Asia | Azerbaijan | Middle SDI | Upper middle income | 0 | 139.25 |
| Central Europe, eastern Europe, and central Asia | Central Asia | Georgia | High-middle SDI | Lower middle income | 0 | 119.60 |
| Central Europe, eastern Europe, and central Asia | Central Asia | Kazakhstan | High-middle SDI | Upper middle income | 3 | 164.22 |
| Central Europe, eastern Europe, and central Asia | Central Asia | Kyrgyzstan | Low-middle SDI | Low income | 3 | 71.41 |
| Central Europe, eastern Europe, and central Asia | Central Asia | Mongolia | Low-middle SDI | Lower middle income | 2 | 95.07 |
| Central Europe, eastern Europe, and central Asia | Central Asia | Tajikistan | Low-middle SDI | Low income | 6 | 55.14 |
| Central Europe, eastern Europe, and central Asia | Central Asia | Turkmenistan | Middle SDI | Upper middle income | 0 | 93.83 |
| Central Europe, eastern Europe, and central Asia | Central Asia | Uzbekistan | Middle SDI | Lower middle income | 1 | 96.76 |
| Central Europe, eastern Europe, and central Asia | Central Europe | Albania | Middle SDI | Lower middle income | 2 | 83.26 |
| Central Europe, eastern Europe, and central Asia | Central Europe | Bosnia and Herzegovina | High-middle SDI | Upper middle income | 1 | 68.10 |
| Central Europe, eastern Europe, and central Asia | Central Europe | Bulgaria | High-middle SDI | Upper middle income | 0 | 212.48 |
| Central Europe, eastern Europe, and central Asia | Central Europe | Croatia | High-middle SDI | High income | 0 | 149.93 |
| Central Europe, eastern Europe, and central Asia | Central Europe | Czechia | High SDI | High income | 0 | 242.32 |
| Central Europe, eastern Europe, and central Asia | Central Europe | Hungary | High-middle SDI | High income | 0 | 180.94 |
| Central Europe, eastern Europe, and central Asia | Central Europe | North Macedonia | High-middle SDI | Upper middle income | 1 | 89.10 |
| Central Europe, eastern Europe, and central Asia | Central Europe | Montenegro | High-middle SDI | Upper middle income | 0 | 141.73 |
| Central Europe, eastern Europe, and central Asia | Central Europe | Poland | High SDI | High income | 0 | 166.63 |
| Central Europe, eastern Europe, and central Asia | Central Europe | Romania | High-middle SDI | Upper middle income | 2 | 150.22 |
| Central Europe, eastern Europe, and central Asia | Central Europe | Serbia | High-middle SDI | Upper middle income | 0 | 81.02 |
| Central Europe, eastern Europe, and central Asia | Central Europe | Slovakia | High SDI | High income | 0 | 234.68 |
| Central Europe, eastern Europe, and central Asia | Central Europe | Slovenia | High SDI | High income | 0 | 237.41 |
| Central Europe, eastern Europe, and central Asia | Eastern Europe | Belarus | High-middle SDI | Upper middle income | 2 | 194.57 |
| Central Europe, eastern Europe, and central Asia | Eastern Europe | Estonia | High SDI | High income | 0 | 193.28 |
| Central Europe, eastern Europe, and central Asia | Eastern Europe | Latvia | High SDI | Upper middle income | 1 | 185.17 |
| Central Europe, eastern Europe, and central Asia | Eastern Europe | Lithuania | High SDI | Upper middle income | 0 | 193.28 |
| Central Europe, eastern Europe, and central Asia | Eastern Europe | Republic of Moldova | High-middle SDI | Lower middle income | 1 | 71.54 |
| Central Europe, eastern Europe, and central Asia | Eastern Europe | Russian Federation | High-middle SDI | Upper middle income | 9 | 235.19 |
| Central Europe, eastern Europe, and central Asia | Eastern Europe | Ukraine | High-middle SDI | Lower middle income | 3 | 204.35 |
| High income | High-income Asia Pacific | Brunei Darussalam | High-middle SDI | High income | 0 | 178.22 |
| High income | High-income Asia Pacific | Japan | High SDI | High income | 1 | 290.97 |
| High income | High-income Asia Pacific | Republic of Korea | High SDI | High income | 3 | 115.30 |
| High income | High-income Asia Pacific | Singapore | High SDI | High income | 3 | 222.19 |
| High income | Australasia | Australia | High SDI | High income | 1 | 425.36 |
| High income | Australasia | New Zealand | High SDI | High income | 0 | 392.62 |
| High income | Western Europe | Austria | High SDI | High income | 0 | 286.01 |
| High income | Western Europe | Belgium | High SDI | High income | 0 | 306.05 |
| High income | Western Europe | Cyprus | High SDI | High income | 1 | 151.98 |
| High income | Western Europe | Denmark | High SDI | High income | 0 | 618.24 |
| High income | Western Europe | Finland | High SDI | High income | 0 | 473.96 |
| High income | Western Europe | France | High SDI | High income | 1 | 332.09 |
| High income | Western Europe | Germany | High SDI | High income | 1 | 356.10 |
| High income | Western Europe | Greece | High-middle SDI | High income | 0 | 141.79 |
| High income | Western Europe | Iceland | High SDI | High income | 0 | 427.13 |
| High income | Western Europe | Ireland | High SDI | High income | 1 | 273.54 |
| High income | Western Europe | Israel | High-middle SDI | High income | 1 | 250.89 |
| High income | Western Europe | Italy | High-middle SDI | High income | 1 | 198.98 |
| High income | Western Europe | Luxembourg | High SDI | High income | 0 | 232.45 |
| High income | Western Europe | Malta | High-middle SDI | High income | 0 | 195.90 |
| High income | Western Europe | Netherlands | High SDI | High income | 1 | 479.19 |
| High income | Western Europe | Norway | High SDI | High income | 0 | 599.94 |
| High income | Western Europe | Portugal | High-middle SDI | High income | 0 | 175.43 |
| High income | Western Europe | Spain | High-middle SDI | High income | 2 | 214.85 |
| High income | Western Europe | Sweden | High SDI | High income | 1 | 649.06 |
| High income | Western Europe | Switzerland | High SDI | High income | 0 | 459.02 |
| High income | Western Europe | United Kingdom | High SDI | High income | 1 | 347.52 |
| High income | High-income North America | Canada | High SDI | High income | 4 | 421.20 |
| High income | High-income North America | United States of America | High SDI | High income | 4 | 371.34 |
| Latin America and Caribbean | Southern Latin America | Argentina | High-middle SDI | Upper middle income | 2 | 179.74 |
| Latin America and Caribbean | Southern Latin America | Chile | High-middle SDI | Upper middle income | 1 | 122.90 |
| Latin America and Caribbean | Southern Latin America | Uruguay | High-middle SDI | Upper middle income | 0 | 184.18 |
| Latin America and Caribbean | Caribbean | Antigua and Barbuda | High-middle SDI | Upper middle income | 0 | 154.66 |
| Latin America and Caribbean | Caribbean | Bahamas | High-middle SDI | High income | 0 | 262.33 |
| Latin America and Caribbean | Caribbean | Barbados | High-middle SDI | High income | 0 | 176.82 |
| Latin America and Caribbean | Caribbean | Belize | Low-middle SDI | Lower middle income | 0 | 54.79 |
| Latin America and Caribbean | Caribbean | Cuba | Middle SDI | Upper middle income | 2 | 256.97 |
| Latin America and Caribbean | Caribbean | Dominica | High-middle SDI | Upper middle income | 0 | 82.80 |
| Latin America and Caribbean | Caribbean | Dominican Republic | Middle SDI | Upper middle income | 8 | 71.82 |
| Latin America and Caribbean | Caribbean | Grenada | Middle SDI | Upper middle income | 0 | 89.46 |
| Latin America and Caribbean | Caribbean | Guyana | Middle SDI | Lower middle income | 0 | 52.82 |
| Latin America and Caribbean | Caribbean | Haiti | Low SDI | Low income | 7 | 20.82 |
| Latin America and Caribbean | Caribbean | Jamaica | Middle SDI | Upper middle income | 2 | 86.06 |
| Latin America and Caribbean | Caribbean | Saint Lucia | Middle SDI | Upper middle income | 0 | 100.99 |
| Latin America and Caribbean | Caribbean | Saint Vincent and the Grenadines | Middle SDI | Upper middle income | 0 | 72.86 |
| Latin America and Caribbean | Caribbean | Suriname | Middle SDI | Upper middle income | 0 | 141.43 |
| Latin America and Caribbean | Caribbean | Trinidad and Tobago | High-middle SDI | High income | 0 | 103.19 |
| Latin America and Caribbean | Andean Latin America | Bolivia (Plurinational State of) | Low-middle SDI | Lower middle income | 10 | 76.34 |
| Latin America and Caribbean | Andean Latin America | Ecuador | Middle SDI | Upper middle income | 10 | 64.61 |
| Latin America and Caribbean | Andean Latin America | Peru | Middle SDI | Upper middle income | 11 | 86.38 |
| Latin America and Caribbean | Central Latin America | Colombia | Middle SDI | Upper middle income | 6 | 88.74 |
| Latin America and Caribbean | Central Latin America | Costa Rica | Middle SDI | Upper middle income | 3 | 98.19 |
| Latin America and Caribbean | Central Latin America | El Salvador | Low-middle SDI | Lower middle income | 10 | 50.83 |
| Latin America and Caribbean | Central Latin America | Guatemala | Low-middle SDI | Lower middle income | 9 | 48.25 |
| Latin America and Caribbean | Central Latin America | Honduras | Low-middle SDI | Lower middle income | 8 | 32.35 |
| Latin America and Caribbean | Central Latin America | Mexico | Middle SDI | Upper middle income | 3 | 135.61 |
| Latin America and Caribbean | Central Latin America | Nicaragua | Low-middle SDI | Lower middle income | 11 | 39.32 |
| Latin America and Caribbean | Central Latin America | Panama | Middle SDI | Upper middle income | 4 | 103.22 |
| Latin America and Caribbean | Central Latin America | Venezuela (Bolivarian Republic of) | Low-middle SDI | Upper middle income | 5 | 83.30 |
| Latin America and Caribbean | Tropical Latin America | Brazil | Middle SDI | Upper middle income | 13 | 106.24 |
| Latin America and Caribbean | Tropical Latin America | Paraguay | Middle SDI | Lower middle income | 8 | 62.16 |
| Latin America and Caribbean | Caribbean | Bermuda | High SDI | High income | 0 | 426.19 |
| Latin America and Caribbean | Caribbean | United States Virgin Islands | High SDI | High income | 0 | 259.80 |
| Latin America and Caribbean | Caribbean | Puerto Rico | High SDI | High income | 0 | 174.38 |
| Latin America and Caribbean | Caribbean | Saint Kitts and Nevis | High-middle SDI | High income | 0 | 170.64 |
| North Africa and Middle East | North Africa and Middle East | Algeria | Middle SDI | Upper middle income | 2 | 34.14 |
| North Africa and Middle East | North Africa and Middle East | Bahrain | High-middle SDI | High income | 0 | 126.84 |
| North Africa and Middle East | North Africa and Middle East | Egypt | Low-middle SDI | Lower middle income | 1 | 48.49 |
| North Africa and Middle East | North Africa and Middle East | Iran (Islamic Republic of) | Middle SDI | Upper middle income | 1 | 39.42 |
| North Africa and Middle East | North Africa and Middle East | Iraq | Middle SDI | Lower middle income | 5 | 30.97 |
| North Africa and Middle East | North Africa and Middle East | Jordan | High-middle SDI | Upper middle income | 0 | 72.11 |
| North Africa and Middle East | North Africa and Middle East | Kuwait | High SDI | High income | 0 | 185.58 |
| North Africa and Middle East | North Africa and Middle East | Lebanon | High-middle SDI | Upper middle income | 0 | 61.06 |
| North Africa and Middle East | North Africa and Middle East | Libya | High-middle SDI | Upper middle income | 0 | 56.51 |
| North Africa and Middle East | North Africa and Middle East | Morocco | Low-middle SDI | Lower middle income | 0 | 21.31 |
| North Africa and Middle East | North Africa and Middle East | Palestine | Middle SDI | Lower middle income | 0 | 21.09 |
| North Africa and Middle East | North Africa and Middle East | Oman | High-middle SDI | High income | 0 | 81.47 |
| North Africa and Middle East | North Africa and Middle East | Qatar | High SDI | High income | 0 | 207.58 |
| North Africa and Middle East | North Africa and Middle East | Saudi Arabia | High SDI | High income | 3 | 86.66 |
| North Africa and Middle East | North Africa and Middle East | Syrian Arab Republic | Middle SDI | Lower middle income | 0 | 26.06 |
| North Africa and Middle East | North Africa and Middle East | Tunisia | Middle SDI | Upper middle income | 0 | 38.44 |
| North Africa and Middle East | North Africa and Middle East | Turkey | High-middle SDI | Upper middle income | 1 | 46.76 |
| North Africa and Middle East | North Africa and Middle East | United Arab Emirates | High SDI | High income | 0 | 224.85 |
| North Africa and Middle East | North Africa and Middle East | Yemen | Low SDI | Lower middle income | 8 | 13.34 |
| North Africa and Middle East | North Africa and Middle East | Afghanistan | Low SDI | Low income | 20 | 11.17 |
| North Africa and Middle East | North Africa and Middle East | Sudan | Low-middle SDI | Lower middle income | 33 | 17.04 |
| South Asia | South Asia | Bangladesh | Low-middle SDI | Low income | 18 | 25.97 |
| South Asia | South Asia | Bhutan | Low-middle SDI | Lower middle income | 1 | 52.12 |
| South Asia | South Asia | India | Low-middle SDI | Lower middle income | 40 | 27.83 |
| South Asia | South Asia | Nepal | Low SDI | Low income | 17 | 36.75 |
| South Asia | South Asia | Pakistan | Low-middle SDI | Lower middle income | 11 | 27.37 |
| Southeast Asia, east Asia, and Oceania | East Asia | China | High-middle SDI | Upper middle income | 4 | 77.18 |
| Southeast Asia, east Asia, and Oceania | Southeast Asia | Cambodia | Low-middle SDI | Low income | 8 | 37.72 |
| Southeast Asia, east Asia, and Oceania | Southeast Asia | Indonesia | Middle SDI | Lower middle income | 22 | 51.27 |
| Southeast Asia, east Asia, and Oceania | Southeast Asia | Lao People's Democratic Republic | Low-middle SDI | Lower middle income | 6 | 31.26 |
| Southeast Asia, east Asia, and Oceania | Southeast Asia | Malaysia | High-middle SDI | Upper middle income | 10 | 89.43 |
| Southeast Asia, east Asia, and Oceania | Southeast Asia | Maldives | Middle SDI | Upper middle income | 1 | 80.65 |
| Southeast Asia, east Asia, and Oceania | Southeast Asia | Myanmar | Low-middle SDI | Low income | 2 | 26.72 |
| Southeast Asia, east Asia, and Oceania | Southeast Asia | Philippines | Middle SDI | Lower middle income | 17 | 51.97 |
| Southeast Asia, east Asia, and Oceania | Southeast Asia | Sri Lanka | Middle SDI | Lower middle income | 8 | 48.05 |
| Southeast Asia, east Asia, and Oceania | Southeast Asia | Thailand | Middle SDI | Upper middle income | 4 | 103.39 |
| Southeast Asia, east Asia, and Oceania | Southeast Asia | Timor-Leste | Low SDI | Lower middle income | 2 | 15.15 |
| Southeast Asia, east Asia, and Oceania | Southeast Asia | Viet Nam | Middle SDI | Lower middle income | 8 | 44.87 |
| Southeast Asia, east Asia, and Oceania | Oceania | Fiji | Middle SDI | Lower middle income | 1 | 55.46 |
| Southeast Asia, east Asia, and Oceania | Oceania | Kiribati | Low-middle SDI | Lower middle income | 0 | 32.11 |
| Southeast Asia, east Asia, and Oceania | Oceania | Marshall Islands | Low-middle SDI | Lower middle income | 1 | 42.95 |
| Southeast Asia, east Asia, and Oceania | Oceania | Micronesia (Federated States of) | Low-middle SDI | Lower middle income | 1 | 40.25 |
| Southeast Asia, east Asia, and Oceania | Oceania | Papua New Guinea | Low SDI | Lower middle income | 7 | 19.00 |
| Southeast Asia, east Asia, and Oceania | Oceania | Samoa | Low-middle SDI | Lower middle income | 1 | 29.81 |
| Southeast Asia, east Asia, and Oceania | Oceania | Solomon Islands | Low SDI | Lower middle income | 2 | 24.30 |
| Southeast Asia, east Asia, and Oceania | Oceania | Tonga | Middle SDI | Lower middle income | 2 | 36.87 |
| Southeast Asia, east Asia, and Oceania | Oceania | Vanuatu | Low-middle SDI | Lower middle income | 1 | 25.57 |
| Southeast Asia, east Asia, and Oceania | Southeast Asia | Mauritius | High-middle SDI | Upper middle income | 1 | 72.15 |
| Southeast Asia, east Asia, and Oceania | Southeast Asia | Seychelles | High-middle SDI | Upper middle income | 2 | 114.70 |
| Southeast Asia, east Asia, and Oceania | Oceania | American Samoa | High-middle SDI | Upper middle income | 0 | 93.62 |
| Southeast Asia, east Asia, and Oceania | Oceania | Guam | High-middle SDI | High income | 0 | 194.35 |
| Southeast Asia, east Asia, and Oceania | Oceania | Northern Mariana Islands | High-middle SDI | High income | 0 | 183.04 |
| Southeast Asia, east Asia, and Oceania | Oceania | Palau | High-middle SDI | Upper middle income | 1 | 136.98 |
| Southeast Asia, east Asia, and Oceania | Oceania | Tuvalu | Low-middle SDI | Upper middle income | 0 | 49.37 |
| Sub-Saharan Africa | Central Sub-Saharan Africa | Angola | Low SDI | Upper middle income | 16 | 35.30 |
| Sub-Saharan Africa | Central Sub-Saharan Africa | Central African Republic | Low SDI | Low income | 12 | 25.12 |
| Sub-Saharan Africa | Central Sub-Saharan Africa | Congo | Low-middle SDI | Lower middle income | 18 | 48.27 |
| Sub-Saharan Africa | Central Sub-Saharan Africa | Democratic Republic of the Congo | Low SDI | Low income | 72 | 42.95 |
| Sub-Saharan Africa | Central Sub-Saharan Africa | Equatorial Guinea | Middle SDI | High income | 1 | 88.80 |
| Sub-Saharan Africa | Central Sub-Saharan Africa | Gabon | Middle SDI | Upper middle income | 7 | 73.50 |
| Sub-Saharan Africa | Eastern Sub-Saharan Africa | Burundi | Low SDI | Low income | 14 | 19.39 |
| Sub-Saharan Africa | Eastern Sub-Saharan Africa | Comoros | Low-middle SDI | Low income | 5 | 33.70 |
| Sub-Saharan Africa | Eastern Sub-Saharan Africa | Djibouti | Low-middle SDI | Lower middle income | 5 | 18.07 |
| Sub-Saharan Africa | Eastern Sub-Saharan Africa | Eritrea | Low SDI | Low income | 0 | 20.71 |
| Sub-Saharan Africa | Eastern Sub-Saharan Africa | Ethiopia | Low SDI | Low income | 22 | 10.11 |
| Sub-Saharan Africa | Eastern Sub-Saharan Africa | Kenya | Low-middle SDI | Low income | 35 | 36.38 |
| Sub-Saharan Africa | Eastern Sub-Saharan Africa | Madagascar | Low SDI | Low income | 8 | 26.89 |
| Sub-Saharan Africa | Eastern Sub-Saharan Africa | Malawi | Low SDI | Low income | 14 | 20.99 |
| Sub-Saharan Africa | Eastern Sub-Saharan Africa | Mozambique | Low SDI | Low income | 27 | 15.72 |
| Sub-Saharan Africa | Eastern Sub-Saharan Africa | Rwanda | Low SDI | Low income | 10 | 16.40 |
| Sub-Saharan Africa | Eastern Sub-Saharan Africa | Somalia | Low SDI | Low income | 25 | 10.08 |
| Sub-Saharan Africa | Eastern Sub-Saharan Africa | United Republic of Tanzania | Low SDI | Low income | 27 | 34.51 |
| Sub-Saharan Africa | Eastern Sub-Saharan Africa | Uganda | Low SDI | Low income | 35 | 24.00 |
| Sub-Saharan Africa | Eastern Sub-Saharan Africa | Zambia | Low-middle SDI | Lower middle income | 18 | 31.81 |
| Sub-Saharan Africa | Southern Sub-Saharan Africa | Botswana | Middle SDI | Upper middle income | 2 | 61.45 |
| Sub-Saharan Africa | Southern Sub-Saharan Africa | Lesotho | Low-middle SDI | Lower middle income | 2 | 30.36 |
| Sub-Saharan Africa | Southern Sub-Saharan Africa | Namibia | Low-middle SDI | Upper middle income | 6 | 36.65 |
| Sub-Saharan Africa | Southern Sub-Saharan Africa | South Africa | Middle SDI | Upper middle income | 6 | 78.39 |
| Sub-Saharan Africa | Southern Sub-Saharan Africa | Eswatini | Low-middle SDI | Lower middle income | 3 | 34.32 |
| Sub-Saharan Africa | Southern Sub-Saharan Africa | Zimbabwe | Low-middle SDI | Low income | 24 | 44.54 |
| Sub-Saharan Africa | Western Sub-Saharan Africa | Benin | Low SDI | Low income | 22 | 20.48 |
| Sub-Saharan Africa | Western Sub-Saharan Africa | Burkina Faso | Low SDI | Low income | 18 | 16.17 |
| Sub-Saharan Africa | Western Sub-Saharan Africa | Cameroon | Low-middle SDI | Lower middle income | 22 | 51.26 |
| Sub-Saharan Africa | Western Sub-Saharan Africa | Cabo Verde | Low-middle SDI | Lower middle income | 2 | 32.97 |
| Sub-Saharan Africa | Western Sub-Saharan Africa | Chad | Low SDI | Low income | 21 | 13.85 |
| Sub-Saharan Africa | Western Sub-Saharan Africa | Coted'Ivoire | Low SDI | Lower middle income | 13 | 27.46 |
| Sub-Saharan Africa | Western Sub-Saharan Africa | Gambia | Low SDI | Low income | 3 | 23.44 |
| Sub-Saharan Africa | Western Sub-Saharan Africa | Ghana | Low-middle SDI | Lower middle income | 18 | 39.20 |
| Sub-Saharan Africa | Western Sub-Saharan Africa | Guinea | Low SDI | Low income | 15 | 10.48 |
| Sub-Saharan Africa | Western Sub-Saharan Africa | Guinea-Bissau | Low SDI | Low income | 7 | 20.79 |
| Sub-Saharan Africa | Western Sub-Saharan Africa | Liberia | Low SDI | Low income | 11 | 22.55 |
| Sub-Saharan Africa | Western Sub-Saharan Africa | Mali | Low SDI | Low income | 11 | 17.04 |
| Sub-Saharan Africa | Western Sub-Saharan Africa | Mauritania | Low-middle SDI | Low income | 3 | 21.95 |
| Sub-Saharan Africa | Western Sub-Saharan Africa | Niger | Low SDI | Low income | 38 | 12.93 |
| Sub-Saharan Africa | Western Sub-Saharan Africa | Nigeria | Low-middle SDI | Lower middle income | 55 | 28.97 |
| Sub-Saharan Africa | Western Sub-Saharan Africa | Sao Tome and Principe | Low-middle SDI | Lower middle income | 1 | 21.00 |
| Sub-Saharan Africa | Western Sub-Saharan Africa | Senegal | Low SDI | Lower middle income | 8 | 19.74 |
| Sub-Saharan Africa | Western Sub-Saharan Africa | Sierra Leone | Low SDI | Low income | 15 | 22.10 |
| Sub-Saharan Africa | Western Sub-Saharan Africa | Togo | Low SDI | Low income | 11 | 26.43 |
| Sub-Saharan Africa | Eastern Sub-Saharan Africa | South Sudan | Low SDI | Low income | 7 | 18.79 |

Abbreviations: CHWs, Community Health Workers; GBD, global burden of disease; HRHs, human resources for health; SDI, sociodemographic index.

## Fig A. Region-specific Lorenz curves and Gini indices for epidemics and all HRHs.

(A) Lorenz curve of epidemics; (B) Lorenz curve of all HRHs; (C) Gini index of epidemics; (D) Gini index of all HRHs. Abbreviations: HRH, human resources for health.

## Fig B. Association between epidemics and cadre-specific HRHs at different lags (0-5 year).

Note: Generalized estimating equation (GEE) models were used to calculate the effect sizes. Points represent effect sizes, and error bars represent 95% confidence intervals. The p values for coefficients of the GEE models were derived from Wald tests. Abbreviations: CHWs, Community Health Workers; HRHs, human resources for health.

## Fig C. Association between epidemics and cadre-specific HRH density by SDI level, World Bank income groups, and GBD super regions.

Note: Generalized estimating equation (GEE) models were used to calculate the effect sizes. Points represent effect sizes, and error bars represent 95% confidence intervals. The p values for coefficients of the GEE models were derived from Wald tests. Abbreviations: CHWs, Community Health Workers; GBD, global burden of disease; HRHs, human resources for health; SDI, sociodemographic index.

## Table D. Global annual average cadre-specific HRH losses attributable to epidemics from 1990 to 2019 among all countries and territories.

| Cadres | Annual AP (95%CI) | Annual AF (95%CI) |
| --- | --- | --- |
| All health workers | 17549 (5661, 29437) | 2.57 (0.83, 4.31) |
| Aides & Emergency Medical Workers | 3162 (-1486, 7810) | 3.35 (-1.57, 8.28) |
| Audiologists and Counsellors | 467 (213, 722) | 2.46 (1.12, 3.79) |
| Dentistry personnel | 410 (-202, 1022) | 1.36 (-0.67, 3.38) |
| Dieticians & Nutritionists | 171 (44, 297) | 2.24 (0.58, 3.89) |
| Environmental Health Officers | 695 (146, 1245) | 5.27 (1.10, 9.44) |
| Medical Assistants & CHWs | 851 (274, 1428) | 2.89 (0.93, 4.86) |
| Medical Lab Technicians | 224 (-53, 502) | 0.87 (-0.20, 1.94) |
| Nursing and midwifery personnel | 6125 (2251, 9998) | 3.00 (1.10, 4.90) |
| Optometrists | 154 (47, 260) | 2.74 (0.83, 4.64) |
| Personal Care Workers | 1025 (279, 1771) | 1.73 (0.47, 2.98) |
| Pharmaceutical personnel | 854 (147, 1562) | 2.60 (0.45, 4.76) |
| Physicians | 1478 (125, 2832) | 1.64 (0.14, 3.14) |
| Psychologists | 263 (-153, 679) | 1.72 (-1.00, 4.45) |
| Radiographers | 210 (37, 383) | 1.15 (0.20, 2.09) |
| Traditional & Complementary Practitioners | 613 (-196, 1422) | 3.59 (-1.15, 8.33) |

Abbreviations: CHWs, Community Health Workers; HRHs, human resources for health; AF, Attributable fraction; AP, Attributable person.

## Table E. Annual average HRH losses attributable to epidemics from 1990 to 2019 among countries and territories

| Countries and territories | Annual AP (95%CI) | Annual AF (95%CI) |
| --- | --- | --- |
| Afghanistan | 68 (22, 113) | 30.10 (9.71, 50.49) |
| Albania | 1 (0, 2) | 0.50 (0.16, 0.84) |
| Algeria | 11 (4, 19) | 0.91 (0.29, 1.53) |
| American Samoa | 0 (0, 0) | 0.00 (0.00, 0.00) |
| Andorra | 0 (0, 0) | 0.00 (0.00, 0.00) |
| Angola | 61 (20, 102) | 8.60 (2.77, 14.43) |
| Antigua and Barbuda | 0 (0, 0) | 0.00 (0.00, 0.00) |
| Argentina | 15 (5, 25) | 0.21 (0.07, 0.35) |
| Armenia | 0 (0, 0) | 0.00 (0.00, 0.00) |
| Australia | 5 (2, 8) | 0.05 (0.02, 0.09) |
| Austria | 0 (0, 0) | 0.00 (0.00, 0.00) |
| Azerbaijan | 0 (0, 0) | 0.00 (0.00, 0.00) |
| Bahamas | 0 (0, 0) | 0.00 (0.00, 0.00) |
| Bahrain | 0 (0, 0) | 0.00 (0.00, 0.00) |
| Bangladesh | 463 (149, 776) | 12.28 (3.96, 20.61) |
| Barbados | 0 (0, 0) | 0.00 (0.00, 0.00) |
| Belarus | 4 (1, 7) | 0.21 (0.07, 0.36) |
| Belgium | 0 (0, 0) | 0.00 (0.00, 0.00) |
| Belize | 0 (0, 0) | 0.00 (0.00, 0.00) |
| Benin | 34 (11, 57) | 19.24 (6.20, 32.27) |
| Bermuda | 0 (0, 0) | 0.00 (0.00, 0.00) |
| Bhutan | 0 (0, 0) | 0.33 (0.11, 0.56) |
| Bolivia (Plurinational State of) | 18 (6, 30) | 2.38 (0.77, 3.99) |
| Bosnia and Herzegovina | 1 (0, 1) | 0.30 (0.10, 0.50) |
| Botswana | 1 (0, 1) | 0.63 (0.20, 1.05) |
| Brazil | 472 (152, 792) | 2.29 (0.74, 3.85) |
| Brunei Darussalam | 0 (0, 0) | 0.00 (0.00, 0.00) |
| Bulgaria | 0 (0, 0) | 0.00 (0.00, 0.00) |
| Burkina Faso | 51 (17, 86) | 20.53 (6.62, 34.44) |
| Burundi | 20 (6, 33) | 12.08 (3.90, 20.26) |
| Cabo Verde | 0 (0, 0) | 1.10 (0.35, 1.85) |
| Cambodia | 20 (6, 33) | 3.62 (1.17, 6.07) |
| Cameroon | 78 (25, 131) | 7.62 (2.46, 12.77) |
| Canada | 23 (8, 39) | 0.17 (0.05, 0.28) |
| Central African Republic | 9 (3, 15) | 8.50 (2.74, 14.26) |
| Chad | 45 (15, 76) | 30.69 (9.90, 51.47) |
| Chile | 3 (1, 5) | 0.13 (0.04, 0.22) |
| China | 1004 (324, 1683) | 0.96 (0.31, 1.61) |
| Colombia | 41 (13, 70) | 1.09 (0.35, 1.83) |
| Comoros | 1 (0, 1) | 2.80 (0.90, 4.70) |
| Congo | 11 (4, 19) | 5.88 (1.90, 9.86) |
| Cook Islands | 0 (0, 0) | 0.48 (0.15, 0.80) |
| Costa Rica | 2 (1, 3) | 0.39 (0.13, 0.65) |
| Coted'Ivoire | 49 (16, 82) | 8.97 (2.89, 15.05) |
| Croatia | 0 (0, 0) | 0.00 (0.00, 0.00) |
| Cuba | 4 (1, 7) | 0.15 (0.05, 0.25) |
| Cyprus | 0 (0, 0) | 0.11 (0.03, 0.18) |
| Czechia | 0 (0, 0) | 0.00 (0.00, 0.00) |
| Democratic People's Republic of Korea | 15 (5, 25) | 2.68 (0.86, 4.49) |
| Democratic Republic of the Congo | 825 (266, 1384) | 30.48 (9.83, 51.12) |
| Denmark | 0 (0, 0) | 0.00 (0.00, 0.00) |
| Djibouti | 1 (0, 1) | 4.14 (1.34, 6.94) |
| Dominica | 0 (0, 0) | 0.00 (0.00, 0.00) |
| Dominican Republic | 13 (4, 21) | 1.83 (0.59, 3.07) |
| Ecuador | 25 (8, 42) | 2.72 (0.88, 4.57) |
| Egypt | 15 (5, 25) | 0.38 (0.12, 0.64) |
| El Salvador | 10 (3, 17) | 3.36 (1.08, 5.63) |
| Equatorial Guinea | 0 (0, 0) | 0.17 (0.06, 0.29) |
| Eritrea | 0 (0, 0) | 0.00 (0.00, 0.00) |
| Estonia | 0 (0, 0) | 0.00 (0.00, 0.00) |
| Eswatini | 1 (0, 1) | 1.56 (0.50, 2.62) |
| Ethiopia | 315 (102, 528) | 37.12 (11.97, 62.26) |
| Fiji | 0 (0, 0) | 0.00 (0.00, 0.00) |
| Finland | 0 (0, 0) | 0.00 (0.00, 0.00) |
| France | 12 (4, 19) | 0.06 (0.02, 0.09) |
| Gabon | 2 (1, 3) | 1.76 (0.57, 2.95) |
| Gambia | 1 (0, 2) | 2.44 (0.79, 4.09) |
| Georgia | 0 (0, 0) | 0.00 (0.00, 0.00) |
| Germany | 16 (5, 27) | 0.06 (0.02, 0.09) |
| Ghana | 83 (27, 139) | 8.87 (2.86, 14.88) |
| Greece | 0 (0, 0) | 0.00 (0.00, 0.00) |
| Greenland | 0 (0, 0) | 0.00 (0.00, 0.00) |
| Grenada | 0 (0, 0) | 0.00 (0.00, 0.00) |
| Guam | 0 (0, 0) | 0.00 (0.00, 0.00) |
| Guatemala | 18 (6, 29) | 2.80 (0.90, 4.70) |
| Guinea | 28 (9, 47) | 27.93 (9.01, 46.85) |
| Guinea-Bissau | 2 (1, 3) | 5.83 (1.88, 9.78) |
| Guyana | 0 (0, 0) | 0.00 (0.00, 0.00) |
| Haiti | 15 (5, 25) | 7.45 (2.40, 12.49) |
| Honduras | 10 (3, 16) | 3.93 (1.27, 6.59) |
| Hungary | 0 (0, 0) | 0.00 (0.00, 0.00) |
| Iceland | 0 (0, 0) | 0.00 (0.00, 0.00) |
| India | 7907 (2550, 13263) | 23.84 (7.69, 40.00) |
| Indonesia | 915 (295, 1535) | 7.48 (2.41, 12.54) |
| Iran (Islamic Republic of) | 13 (4, 22) | 0.45 (0.14, 0.75) |
| Iraq | 30 (10, 50) | 3.05 (0.98, 5.12) |
| Ireland | 1 (0, 1) | 0.06 (0.02, 0.11) |
| Israel | 1 (0, 2) | 0.07 (0.02, 0.11) |
| Italy | 11 (4, 19) | 0.10 (0.03, 0.16) |
| Jamaica | 1 (0, 2) | 0.43 (0.14, 0.73) |
| Japan | 25 (8, 43) | 0.07 (0.02, 0.11) |
| Jordan | 0 (0, 0) | 0.00 (0.00, 0.00) |
| Kazakhstan | 9 (3, 15) | 0.33 (0.11, 0.55) |
| Kenya | 236 (76, 397) | 17.00 (5.48, 28.51) |
| Kiribati | 0 (0, 0) | 0.00 (0.00, 0.00) |
| Kuwait | 0 (0, 0) | 0.00 (0.00, 0.00) |
| Kyrgyzstan | 3 (1, 5) | 0.78 (0.25, 1.31) |
| Lao People's Democratic Republic | 6 (2, 11) | 3.27 (1.06, 5.49) |
| Latvia | 0 (0, 1) | 0.11 (0.04, 0.19) |
| Lebanon | 0 (0, 0) | 0.00 (0.00, 0.00) |
| Lesotho | 1 (0, 1) | 1.26 (0.41, 2.12) |
| Liberia | 7 (2, 11) | 7.40 (2.39, 12.42) |
| Libya | 0 (0, 0) | 0.00 (0.00, 0.00) |
| Lithuania | 0 (0, 0) | 0.00 (0.00, 0.00) |
| Luxembourg | 0 (0, 0) | 0.00 (0.00, 0.00) |
| Madagascar | 34 (11, 56) | 6.37 (2.06, 10.69) |
| Malawi | 35 (11, 60) | 11.86 (3.83, 19.89) |
| Malaysia | 46 (15, 76) | 1.88 (0.61, 3.15) |
| Maldives | 0 (0, 0) | 0.25 (0.08, 0.42) |
| Mali | 30 (10, 50) | 11.88 (3.83, 19.92) |
| Malta | 0 (0, 0) | 0.00 (0.00, 0.00) |
| Marshall Islands | 0 (0, 0) | 0.45 (0.15, 0.75) |
| Mauritania | 2 (1, 3) | 2.33 (0.75, 3.90) |
| Mauritius | 0 (0, 0) | 0.27 (0.09, 0.46) |
| Mexico | 59 (19, 99) | 0.39 (0.13, 0.65) |
| Micronesia (Federated States of) | 0 (0, 0) | 0.50 (0.16, 0.84) |
| Monaco | 0 (0, 0) | 0.00 (0.00, 0.00) |
| Mongolia | 1 (0, 2) | 0.39 (0.12, 0.65) |
| Montenegro | 0 (0, 0) | 0.00 (0.00, 0.00) |
| Morocco | 0 (0, 0) | 0.00 (0.00, 0.00) |
| Mozambique | 102 (33, 172) | 30.06 (9.70, 50.42) |
| Myanmar | 20 (7, 34) | 1.49 (0.48, 2.50) |
| Namibia | 2 (1, 4) | 3.20 (1.03, 5.36) |
| Nauru | 0 (0, 0) | 0.00 (0.00, 0.00) |
| Nepal | 81 (26, 135) | 8.19 (2.64, 13.73) |
| Netherlands | 3 (1, 5) | 0.04 (0.01, 0.07) |
| New Zealand | 0 (0, 0) | 0.00 (0.00, 0.00) |
| Nicaragua | 10 (3, 17) | 4.48 (1.45, 7.52) |
| Niger | 110 (36, 185) | 57.06 (18.41, 95.72) |
| Nigeria | 1521 (491, 2551) | 33.59 (10.83, 56.34) |
| Niue | 0 (0, 0) | 0.24 (0.08, 0.40) |
| North Macedonia | 0 (0, 1) | 0.22 (0.07, 0.36) |
| Northern Mariana Islands | 0 (0, 0) | 0.00 (0.00, 0.00) |
| Norway | 0 (0, 0) | 0.00 (0.00, 0.00) |
| Oman | 0 (0, 0) | 0.00 (0.00, 0.00) |
| Pakistan | 308 (99, 516) | 6.49 (2.09, 10.89) |
| Palau | 0 (0, 0) | 0.14 (0.05, 0.23) |
| Palestine | 0 (0, 0) | 0.00 (0.00, 0.00) |
| Panama | 2 (1, 4) | 0.61 (0.20, 1.02) |
| Papua New Guinea | 9 (3, 15) | 6.92 (2.23, 11.60) |
| Paraguay | 10 (3, 16) | 2.58 (0.83, 4.33) |
| Peru | 58 (19, 96) | 2.32 (0.75, 3.89) |
| Philippines | 263 (85, 441) | 5.66 (1.82, 9.49) |
| Poland | 0 (0, 0) | 0.00 (0.00, 0.00) |
| Portugal | 0 (0, 0) | 0.00 (0.00, 0.00) |
| Puerto Rico | 0 (0, 0) | 0.00 (0.00, 0.00) |
| Qatar | 0 (0, 0) | 0.00 (0.00, 0.00) |
| Republic of Korea | 29 (9, 48) | 0.50 (0.16, 0.84) |
| Republic of Moldova | 1 (0, 1) | 0.29 (0.09, 0.49) |
| Romania | 9 (3, 15) | 0.28 (0.09, 0.47) |
| Russian Federation | 266 (86, 446) | 0.76 (0.25, 1.28) |
| Rwanda | 16 (5, 28) | 9.76 (3.15, 16.38) |
| Saint Kitts and Nevis | 0 (0, 0) | 0.00 (0.00, 0.00) |
| Saint Lucia | 0 (0, 0) | 0.00 (0.00, 0.00) |
| Saint Vincent and the Grenadines | 0 (0, 0) | 0.00 (0.00, 0.00) |
| Samoa | 0 (0, 0) | 0.00 (0.00, 0.00) |
| San Marino | 0 (0, 0) | 0.00 (0.00, 0.00) |
| Sao Tome and Principe | 0 (0, 0) | 0.92 (0.30, 1.54) |
| Saudi Arabia | 13 (4, 21) | 0.52 (0.17, 0.88) |
| Senegal | 18 (6, 30) | 7.76 (2.50, 13.01) |
| Serbia | 0 (0, 0) | 0.00 (0.00, 0.00) |
| Seychelles | 0 (0, 0) | 0.37 (0.12, 0.61) |
| Sierra Leone | 15 (5, 25) | 11.43 (3.69, 19.18) |
| Singapore | 3 (1, 5) | 0.25 (0.08, 0.42) |
| Slovakia | 0 (0, 0) | 0.00 (0.00, 0.00) |
| Slovenia | 0 (0, 0) | 0.00 (0.00, 0.00) |
| Solomon Islands | 0 (0, 0) | 1.82 (0.59, 3.06) |
| Somalia | 60 (19, 100) | 44.19 (14.26, 74.13) |
| South Africa | 57 (18, 95) | 1.48 (0.48, 2.48) |
| South Sudan | 12 (4, 20) | 7.59 (2.45, 12.73) |
| Spain | 16 (5, 27) | 0.17 (0.05, 0.28) |
| Sri Lanka | 28 (9, 46) | 2.82 (0.91, 4.72) |
| Sudan | 185 (60, 310) | 32.67 (10.54, 54.79) |
| Suriname | 0 (0, 0) | 0.00 (0.00, 0.00) |
| Sweden | 2 (1, 3) | 0.03 (0.01, 0.05) |
| Switzerland | 0 (0, 0) | 0.00 (0.00, 0.00) |
| Syrian Arab Republic | 0 (0, 0) | 0.00 (0.00, 0.00) |
| Taiwan (Province of China) | 0 (0, 0) | 0.00 (0.00, 0.00) |
| Tajikistan | 9 (3, 14) | 2.00 (0.65, 3.36) |
| Thailand | 51 (17, 86) | 0.76 (0.25, 1.28) |
| Timor-Leste | 0 (0, 1) | 2.68 (0.86, 4.50) |
| Togo | 12 (4, 21) | 7.89 (2.54, 13.23) |
| Tokelau | 0 (0, 0) | 0.00 (0.00, 0.00) |
| Tonga | 0 (0, 0) | 0.00 (0.00, 0.00) |
| Trinidad and Tobago | 0 (0, 0) | 0.00 (0.00, 0.00) |
| Tunisia | 0 (0, 0) | 0.00 (0.00, 0.00) |
| Turkey | 14 (5, 24) | 0.42 (0.13, 0.70) |
| Turkmenistan | 0 (0, 0) | 0.00 (0.00, 0.00) |
| Tuvalu | 0 (0, 0) | 0.00 (0.00, 0.00) |
| Uganda | 199 (64, 333) | 28.00 (9.03, 46.97) |
| Ukraine | 30 (10, 51) | 0.31 (0.10, 0.52) |
| United Arab Emirates | 0 (0, 0) | 0.00 (0.00, 0.00) |
| United Kingdom | 12 (4, 20) | 0.05 (0.02, 0.09) |
| United Republic of Tanzania | 191 (62, 321) | 13.03 (4.20, 21.86) |
| United States Virgin Islands | 0 (0, 0) | 0.00 (0.00, 0.00) |
| United States of America | 218 (70, 366) | 0.20 (0.06, 0.33) |
| Uruguay | 0 (0, 0) | 0.00 (0.00, 0.00) |
| Uzbekistan | 5 (2, 8) | 0.18 (0.06, 0.30) |
| Vanuatu | 0 (0, 0) | 0.95 (0.31, 1.59) |
| Venezuela (Bolivarian Republic of) | 22 (7, 36) | 1.00 (0.32, 1.69) |
| Viet Nam | 130 (42, 219) | 3.25 (1.05, 5.45) |
| Yemen | 33 (11, 55) | 10.15 (3.28, 17.03) |
| Zambia | 40 (13, 68) | 9.78 (3.16, 16.41) |
| Zimbabwe | 60 (19, 101) | 10.74 (3.47, 18.02) |

Note: AF, Attributable fraction; AP, Attributable person; HRHs, human resources for health; AF, Attributable fraction; AP, Attributable person.

## Fig D. Association between epidemics occurring after the HRH observation year and HRH density.

Note: Generalized estimating equation (GEE) models were used to calculate the effect sizes. Points represent effect sizes, and error bars represent 95% confidence intervals. The p values for coefficients of the GEE models were derived from Wald tests. Abbreviations: CHWs, Community Health Workers; HRHs, human resources for health.

## Fig E. Association between epidemic intensity (measured by epidemic-related deaths) and HRH density.

Note: Generalized estimating equation (GEE) models were used to calculate the effect sizes. Points represent effect sizes per fold increase in epidemic-related deaths, and error bars represent 95% confidence intervals. The p values for coefficients of the GEE models were derived from Wald tests. Abbreviations: CHWs, Community Health Workers; HRHs, human resources for health.

## Fig F. Association between epidemics and HRH density adjusting for communicable DALYs ratio.

Note: Generalized estimating equation (GEE) models were used to calculate the effect sizes. Points represent effect sizes, and error bars represent 95% confidence intervals. The p values for coefficients of the GEE models were derived from Wald tests. Abbreviations: CHWs, Community Health Workers; DALYs, disability adjusted life years; HRHs, human resources for health.

## Fig G. Association between epidemics and HRH density additionally adjusting for calendar year.

Note: Generalized estimating equation (GEE) models were used to calculate the effect sizes. Points represent effect sizes, and error bars represent 95% confidence intervals. The p values for coefficients of the GEE models were derived from Wald tests. Abbreviations: CHWs, Community Health Workers; HRHs, human resources for health.

## Fig H. Association between epidemics and HRH density using continuous SDI adjustment.

Note: Generalized estimating equation (GEE) models were used to calculate the effect sizes. Points represent effect sizes, and error bars represent 95% confidence intervals. The p values for coefficients of the GEE models were derived from Wald tests. Abbreviations: CHWs, Community Health Workers; HRHs, human resources for health.

## Fig I. Association between epidemics and density of HRH cadres in the sensitivity analyses additionally adjusting GDP per capita.

Note: Generalized estimating equation (GEE) models were used to calculate the effect sizes. Points represent effect sizes, and error bars represent 95% confidence intervals. The p values for coefficients of the GEE models were derived from Wald tests. Abbreviations: CHWs, Community Health Workers; GDP, gross domestic product.

## Table F. Global annual average HRH losses attributable to epidemics using the estimates of GBD super regions, SDI level, and World Bank income groups.

| Group Value | Global (main model) |  |  | GBD SDI quintile |  | World Bank income level | GBD Super Region |
| --- | --- | --- | --- | --- | --- | --- | --- |
|  | Annual AF (95%CI) |  |  | Annual AF (95%CI) |  | Annual AF (95%CI) | Annual AF (95%CI) |
| Global | 2.57 (0.83, 4.31) |  |  | 2.74 (-0.23, 5.70) |  | 2.62 (-0.21, 5.46) | 1.34 (-1.03, 3.70) |
| **GBD Super Region** |  |  |  |  |  |  |  |
| Central Europe, eastern Europe, and central Asia | 0.44 (0.14, 0.74) |  |  | 1.83 (0.73, 2.94) |  | 1.17 (0.45, 1.89) | 1.77 (-0.32, 3.86) |
| High income | 0.12 (0.04, 0.20) |  |  | 0.37 (-0.20, 0.95) |  | 0.64 (0.01, 1.27) | 0.44 (-0.25, 1.13) |
| Latin America and Caribbean | 1.27 (0.41, 2.13) |  |  | 3.30 (1.17, 5.44) |  | 3.48 (1.37, 5.58) | 1.61 (-1.92, 5.13) |
| North Africa and Middle East | 1.77 (0.57, 2.97) |  |  | 1.75 (-0.30, 3.80) |  | 1.79 (-0.21, 3.79) | 1.13 (-1.00, 3.27) |
| South Asia | 20.52 (6.62, 34.41) |  |  | 7.07 (-8.95, 23.09) |  | 13.08 (-5.84, 32.00) | -2.20 (-8.45, 4.04) |
| Southeast Asia, east Asia, and Oceania | 1.78 (0.57, 2.98) |  |  | 6.09 (2.37, 9.80) |  | 2.99 (0.70, 5.28) | 2.38 (-0.30, 5.05) |
| Sub-Saharan Africa | 18.03 (5.82, 30.25) |  |  | 8.36 (-6.91, 23.63) |  | 10.84 (-4.71, 26.38) | 11.17 (-2.30, 24.65) |
| **GBD Region** |  |  |  |  |  |  |  |
| Central Asia | 0.29 (0.09, 0.48) |  |  | 0.62 (0.17, 1.08) |  | 0.39 (0.06, 0.72) | 1.16 (-0.21, 2.53) |
| Central Europe | 0.06 (0.02, 0.10) |  |  | 0.24 (0.10, 0.39) |  | 0.16 (0.06, 0.26) | 0.23 (-0.04, 0.51) |
| Eastern Europe | 0.63 (0.20, 1.05) |  |  | 2.71 (1.10, 4.33) |  | 1.73 (0.69, 2.77) | 2.51 (-0.46, 5.48) |
| Australasia | 0.05 (0.01, 0.08) |  |  | 0.14 (-0.09, 0.36) |  | 0.24 (0.00, 0.48) | 0.17 (-0.09, 0.43) |
| High-income Asia Pacific | 0.13 (0.04, 0.21) |  |  | 0.38 (-0.25, 1.01) |  | 0.67 (0.01, 1.34) | 0.46 (-0.26, 1.19) |
| High-income North America | 0.19 (0.06, 0.32) |  |  | 0.58 (-0.37, 1.53) |  | 1.03 (0.01, 2.04) | 0.71 (-0.40, 1.81) |
| Southern Latin America | 0.18 (0.06, 0.30) |  |  | 0.78 (0.32, 1.24) |  | 0.54 (0.23, 0.85) | 0.23 (-0.27, 0.73) |
| Western Europe | 0.06 (0.02, 0.09) |  |  | 0.20 (-0.03, 0.42) |  | 0.30 (0.00, 0.59) | 0.21 (-0.12, 0.53) |
| Andean Latin America | 2.42 (0.78, 4.06) |  |  | 5.90 (2.00, 9.80) |  | 6.26 (2.38, 10.15) | 3.07 (-3.65, 9.78) |
| Caribbean | 0.63 (0.20, 1.05) |  |  | 1.14 (0.27, 2.00) |  | 1.16 (0.34, 1.98) | 0.80 (-0.95, 2.54) |
| Central Latin America | 0.74 (0.24, 1.25) |  |  | 1.40 (0.36, 2.43) |  | 1.76 (0.62, 2.90) | 0.94 (-1.12, 3.01) |
| Tropical Latin America | 2.30 (0.74, 3.86) |  |  | 6.65 (2.53, 10.77) |  | 6.81 (2.82, 10.80) | 2.91 (-3.47, 9.30) |
| North Africa and Middle East | 1.77 (0.57, 2.97) |  |  | 1.75 (-0.30, 3.80) |  | 1.79 (-0.21, 3.79) | 1.13 (-1.00, 3.27) |
| South Asia | 20.52 (6.62, 34.41) |  |  | 7.07 (-8.95, 23.09) |  | 13.08 (-5.84, 32.00) | -2.20 (-8.45, 4.04) |
| East Asia | 0.95 (0.31, 1.59) |  |  | 4.05 (1.63, 6.47) |  | 2.81 (1.17, 4.46) | 1.27 (-0.16, 2.69) |
| Oceania | 3.49 (1.13, 5.86) |  |  | 1.79 (-1.30, 4.87) |  | 2.28 (-0.98, 5.54) | 4.67 (-0.58, 9.92) |
| Southeast Asia | 4.45 (1.43, 7.46) |  |  | 12.70 (4.77, 20.63) |  | 3.56 (-0.81, 7.92) | 5.95 (-0.74, 12.64) |
| Central Sub-Saharan Africa | 23.21 (7.49, 38.93) |  |  | 11.86 (-8.63, 32.35) |  | 14.49 (-4.92, 33.91) | 14.38 (-2.96, 31.73) |
| Eastern Sub-Saharan Africa | 18.68 (6.03, 31.34) |  |  | 8.81 (-7.25, 24.87) |  | 8.42 (-5.98, 22.82) | 11.58 (-2.39, 25.54) |
| Southern Sub-Saharan Africa | 2.59 (0.84, 4.35) |  |  | 4.01 (0.75, 7.27) |  | 4.43 (1.18, 7.68) | 1.61 (-0.33, 3.54) |
| Western Sub-Saharan Africa | 23.40 (7.55, 39.25) |  |  | 8.77 (-9.94, 27.48) |  | 14.44 (-6.75, 35.63) | 14.50 (-2.99, 31.99) |
| **GBD SDI quintile** |  |  |  |  |  |  |  |
| Low SDI | 19.63 (6.33, 32.93) |  |  | 9.97 (-7.35, 27.29) |  | 10.12 (-5.52, 25.77) | 11.85 (-3.20, 26.90) |
| Low-middle SDI | 17.22 (5.55, 28.88) |  |  | 5.91 (-7.52, 19.33) |  | 11.02 (-4.85, 26.90) | 1.09 (-6.37, 8.55) |
| Middle SDI | 2.38 (0.77, 3.99) |  |  | 6.88 (2.62, 11.13) |  | 3.62 (0.68, 6.57) | 3.06 (-1.42, 7.54) |
| High-middle SDI | 0.69 (0.22, 1.15) |  |  | 2.98 (1.21, 4.76) |  | 2.06 (0.82, 3.31) | 1.36 (-0.24, 2.96) |
| High SDI | 0.12 (0.04, 0.20) |  |  | 0.35 (-0.23, 0.94) |  | 0.63 (0.01, 1.25) | 0.42 (-0.24, 1.08) |
| **World Bank income level** |  |  |  |  |  |  |  |
| Low income | 15.89 (5.12, 26.65) |  |  | 7.37 (-6.21, 20.96) |  | 7.05 (-5.11, 19.21) | 8.30 (-3.11, 19.71) |
| Lower middle income | 12.76 (4.11, 21.40) |  |  | 8.35 (-3.19, 19.89) |  | 8.30 (-3.60, 20.19) | 2.67 (-4.48, 9.83) |
| Upper middle income | 0.96 (0.31, 1.61) |  |  | 3.53 (1.38, 5.67) |  | 2.89 (1.21, 4.57) | 1.54 (-0.64, 3.73) |
| High income | 0.12 (0.04, 0.20) |  |  | 0.36 (-0.19, 0.92) |  | 0.62 (0.01, 1.24) | 0.42 (-0.24, 1.07) |

Note: Abbreviations: AF, Attributable fraction; GBD, global burden of disease; HRHs, human resources for health; SDI, sociodemographic index.

## Fig J. Consistency of annal AF value between main model (global) and estimates by SDI level, World Bank income groups, and GBD super regions.

Note: AF, Attributable fraction; GBD, global burden of disease; SDI, sociodemographic index.

## Fig K. Association between epidemics and the proportion of each HRH cadre.

Note: Generalized estimating equation (GEE) models were used to calculate the effect sizes. Points represent effect sizes, and error bars represent 95% confidence intervals. The p values for coefficients of the GEE models were derived from Wald tests. Abbreviations: CHWs, Community Health Workers; HRHs, human resources for health.
